# Melatonin upregulates photosynthesis, carbohydrate and nitrogen metabolism, and antioxidant system under aluminum stress: a sustainable path to higher strawberry yield and quality

**DOI:** 10.1007/s12298-025-01655-6

**Published:** 2025-10-22

**Authors:** Hend A. Hamed, Marwa T. El-Mahdy, Amany H. A. Abeed

**Affiliations:** 1 Department of Horticulture, Faculty of Agriculture, Sohag University, Sohag, 82524 Egypt; 2 Department of Pomology, Faculty of Agriculture, Assiut University, Assiut, 71526 Egypt; 3 Molecular Biology Research and Studies Institute, Assiut University, Assiut, 71526 Egypt; 4 Department of Botany and Microbiology, Faculty of Science, Assiut University, Assiut, 71516 Egypt

**Keywords:** Aluminum toxicity, Strawberry, Melatonin, Antioxidants, Carbohydrate and nitrogen metabolism, Sustainability

## Abstract

Aluminum (Al) toxicity exhibits a challenge for growing strawberries (*Fragaria* x *ananassa* Duch), impacting their growth and nutritional value. Considerably in this study, we explored how melatonin, an endogenous plant hormone, can help alleviate Al stress in strawberry plants. The current research examined the effects of foliar spraying melatonin (0,50, and 100 ppm) on growth indicators, photosynthetic pigment levels, carbon and nitrogen assimilation, oxidative stress markers, and fruit quality attributes under Al stress (100 µM) in a controlled pot experiment conducted in a greenhouse. The results revealed that exposure to Al stress significantly reduced the adequate growth, as well as the yield and quality of fruits. Melatonin application improved plant growth parameters, especially at a concentration of 100 ppm, enhancing the levels of photosynthetic pigments and boosting carbohydrate and nitrogen metabolism. Moreover, melatonin played a role in reducing stress markers while increasing enzymatic antioxidant activities (catalase, superoxide dismutase, ascorbate peroxidase, glutathione peroxide, glutathione-S-transferase, and phenylalanine ammonia-lyase) and secondary metabolites (proline, ascorbic acid, flavonoids, reduced glutathione, and phytochelatins), while decreasing polyphenol oxidase activity as well as phenolics content, implying a role in ROS scavenging. The results underscore the promise of melatonin as a method to enhance the ability of strawberries to withstand Al toxicity and promote friendly agricultural practices in polluted soils.

## Introduction

In recent decades, the agricultural sector has come across increasing challenges due to environmental pressures, including heavy metal pollution in soil (Uchimiya et al. 2020; Xiang et al. 2020; Li et al. 2025). Among pollutants, aluminum (Al) toxicity is a major hazard to agricultural sustainability and food security all over the world (Wei et al. 2021; Ningombam et al. 2024)). Strawberry plants (*Fragaria ananassa*) are particularly vulnerable, as their production is not only economically valuable but also important for human nutrition (Renai et al. 2021). Several variables affect the quality of strawberries, including sugars, minerals, and bioactive substances, which can affect nutritional value, health benefits, and yield. These components might vary significantly depending on factors such as soil health and pollution with heavy metals (Hamed et al. 2025a). As traditional remediation methods fall short of addressing the pervasive issue of Al toxicity, alternatives need to be examined (Rajendran et al. 2022). In this context, melatonin–an endogenous hormone known for its diverse physiological functions–has emerged as a possible important actor in reducing Al-induced stress in various plant species (Sadak and Bakry 2020; Ren et al. 2022; Ofoe et al. 2023).

Despite Al toxicity in soil being a major concern, it has not been given as much focus as other types of pollutants, and further research is required to understand the particular mechanisms governing melatonin-mediated Al resistance. This issue stems from a variety of factors, including the acidic condition of the soil, which can result in the release of hazardous quantities of Al, forming a stable combination with oxygen and silicate (Matsumot 2000; Ranjan et al. 2021). In areas where irrigation water is rich in salts, the situation might worsen because the salts can displace Al from soil particles, making it more accessible to plants. This displacement happens when the calcium and magnesium ions from the salts bind to soil particles, allowing Al ions to be released into the soil solution (Kar et al. 2021).

The association between Al toxicity and plant physiology is complex, with negative consequences emerging at several levels of cellular organization (Kocjan et al. 2025). When Al ions are abundant in soil, they disturb critical cellular processes by interfering with nutrient intake, impeding root growth, and inducing oxidative stress (Singh et al. 2017; Munyaneza et al. 2024). These perturbations result in decreased growth, lower production, and inadequate plant health (Rahman et al. 2018). Traditional approaches to reducing Al toxicity, such as soil amendment and chelation, are sometimes costly and environmentally unsustainable (Shetty et al. 2021), requiring alternative approaches so they intervene. For instance, adding calcium carbonate or calcium hydroxide to acidic soils can raise the pH, decreasing Al solubility. While effective, this method can be expensive and require repeated applications (Yu et al. 2023). Incorporating organic materials like compost can also help by enhancing soil structure and cation exchange capacity, reducing Al toxicity gradually. Chelating agents such as EDTA can form stable complexes with Al ions, making them less harmful to plants (Orr et al. 2020). However, their use is costly and can raise environmental concerns due to their persistence in the soil (Oviedo and Rodríguez 2003).

Known for its role in circadian rhythms and sleep–wake cycles in mammals, melatonin (Zisapel 2018) has recently gained interest in plant biology due to its pleiotropic effects (Hardeland 2016; Sun et al. 2021; Altaf et al. 2021a). Melatonin has profound effects and strong anti-inflammatory properties in addition to its chronobiotic expression and regulates gene expression and stress-related signaling pathways. A growing trend of research has linked melatonin to various physiological activities, including seed germination (Xiao et al. 2019; Dong et al. 2024), root growth (Altaf et al. 2022), flowering, fruit set (Arnao and Hernández-Ruiz 2020; Yang et al. 2025a, b), and stress responses (Yu et al. 2018; Jahan et al. 2024). Melatonin's capacity to achieve sustainability and reduce heavy metal toxicity in several plant species has recently been discovered (Kul et al. 2019; Jiang et al. 2025; Shehzadi et al. 2025); however, its exact involvement in reducing Al-induced toxicity in strawberry plants remains relatively unknown. Although the understanding of melatonin’s certain function or mechanism has come a long way, there remains much to explore. We developed the concrete hypothesis based on a comprehensive review of existing literature. This suggests that melatonin may have the potential to reduce Al-induced oxidative stress and restore cellular homeostasis in Al-stressed strawberry plants.

This study fills this research gap by examining the ability of melatonin to lessen oxidative damage and restore physiological balance during Al stress, drawing on our perspectives. The use of melatonin as a bio-based, sustainable substitute for conventional, frequently unsustainable chemical treatment methods is what makes this work novel. Therefore, the objectives of this study are: 1: Demonstrate the efficacy of melatonin as a biobased treatment for reducing Al toxicity in strawberries. 2: Emphasize the physiological mechanisms that contribute to melatonin’s protective effects. Using such bio-based substances to minimize the toxicity of heavy metals can help build long-term agricultural strategies that reduce environmental stress while conserving crop production and quality.

## Materials and methods

### Experimental setup

The pot trial took place at Horticulture Department, Agriculture College, Sohag University, located in Sohag Governate, Egypt (26°27′38.2′′, 31°39′52.9′′ E). The experiment ran from mid-November to the end of February in the greenhouse with conditions including natural sunlight of 16/8 h light/dark photoperiod, 21/17 ± 2 °C day/night thermoperiod, and 50–70% relative humidity level. The strawberry variety Festival, being an excellent variety that is widely cultivated in Egypt, was used as the test subject for this experiment. Strawberries at the 3–4 leaf stage with a healthy appearance were transplanted into plastic bags with a 1-L capacity, which were filled with a mixture of peat and perlite in (3:1, v/v) ratio. Plants were irrigated daily using a drip irrigation system, and NPK fertilizer (20:20:20) was applied weekly during the experiment. After 4 weeks of transplanting, to assess the alleviating effect of exogenous melatonin application on Al stress, the strawberry leaves were foliar sprayed with melatonin at 0, 50, and 100 ppm. The spraying was performed three times with equal intervals of two days between each application. Following the melatonin treatments, plants were subjected to Al stress by adding 0 and 100 μM/L Al (NO_3_)_3_ to the irrigation water, with applications spaced one week apart. The control group received an equivalent amount of nutrient solution to sustain consistency across treatments. Ten days after the final Al application, samples were collected for next analyses. The experiment was organized with a factorial arrangement and utilized a fully randomized design replicated 4 times across four blocks. All six treatment combinations were included in each block and assigned randomly to ensure exact statistical analysis.

### Assessment of growth parameters and yield analysis

After carefully pulling out the strawberry plants from the growing containers, they were primarily rinsed under running water to eliminate any soil particles. Next, they were randomly divided into groups according to the subsequent analysis. Several growth parameters including the dry weight of root, crown, and shoot (g) were recorded using a measuring scale, and also the leaf number (per plant) was counted. Samples were desiccated in a hot-air oven set at 70 °C until they registered a stable weight. During the fruit harvest, red mature fruits were sampled, and the fruit number (per plant), average fruit weight (g), and fruit yield (g/plant) were assessed.

### Measurement of physiological and biochemical indicators

Within 12 h of harvest, plants were brought to the laboratory at the Botany and Microbiology Department, Assiut University, Egypt, for required physiological and biochemical analyses.

#### Photosynthetic pigments

Chlorophyll a (Chl a), chlorophyll b (Chl b), and carotenoids were analyzed in strawberry leaves following the scheme of Lichtenthaler (1987) and expressed as mg/g FW. In brief, 50 mg of strawberry tissues were extracted in 5 ml ethyl alcohol (95%). The extraction was allowed to boil for 30 min at 60–70 °C. The optical density (OD) of Chl a, Chl b, and carotenoids was checked via spectrophotometer (Jenway™ Genova spectrophotometer, Bibby Scientific, Staffordshire, UK) at 664.2, 648.6, and 470 nm, respectively. The total content of the three pigments was estimated according to Lichtenthaler (1987) equations as follows:

$$ \begin{aligned} {\text{Chl a}}\left( {{\text{mg}}/{\text{g FW}}} \right)\, = & \,\left( {{13}.{\text{36 A}}_{{{664}.{2}}} } \right) {-} \left( {{5}.{\text{19 A}}_{{{648}.{6}}} } \right). \\ {\text{Chl b }}\left( {{\text{mg}}/{\text{g FW}}} \right)\, = & \,\left( {{27}.{\text{43 A}}_{{{648}.{6}}} } \right) \, {-} \left( {{8}.{\text{12 A}}_{{{664}.{2}}} } \right). \\ {\text{carotenoids}}\left( {{\text{mg}}/{\text{g FW}}} \right)\, = & \,\left( {{1}000{\text{ A}}_{{{47}0}} {-}{ 2}.{13}*{\text{Chl a}}{-}{ 97}.{64}*{\text{Chl b}}} \right)/{2}0{9}. \\ \end{aligned} $$

#### Carbohydrate assimilation

Carbohydrate assimilation was estimated by quantifying glucose (mg/g DW) and sucrose (mg/g DW) according to the methods explained by Halhoul and Kleinberg (1972) and Van Handel (1968), respectively. Protocol adopted as follow; 0.05 gm dry tissue was extracted with 5 ml distilled water in boiling water bath for 2 h, then centrifuged and completed to definite volume. Anthrone reagent was prepared by dissolving 150 mg anthrone in 100 ml of 26.2 N sulfuric acid (analytical grade) at room temperature. After all anthrone particles had dissolved, the reagent was stored at 4 °C for at least 1 h prior to use. For glucose estimation, chilled anthrone reagent (1.5 ml) was added to each sample and mixed immediately and vigorously on a vortex mixer. After 10 to 15 min standing at room temperature, color was developed by incubating the tubes in a water bath at 80 °C for 30 min. Tubes were transferred to cracked ice to stop the reaction. The optical density was then measured at 620 nm in Unico UV-2100 spectrophotometer. For sucrose estimation; 0.1 ml 30% aqueous KOH was added to all tubes and keep at 100 °C for 10 min. When the tubes have cooled to room temperature, when the tubes have cooled to room temperature, add 3 ml anthrone reagent and keep at 40 °C for 10–15 min. Measure the optical density at 620. Starch analysis (mg/g DW) was conducted as clarified by Fales (1951) and Schlegel (1956). 0.05 gm dry tissue was mixed with 10 ml HCl (4 N) in test tubes, heated in boiling water bath for 1 h. Finally, this solution was cooled, filtered in sterilized vials and kept in refrigerator until use. 0.1 ml of prepared solution and 4.5 ml anthrone- sulphuric acid reagent were thoroughly mixed. The last mixture was boiled in water bath for 7 min and directly cooled under tap water. The absorbance of the developed blue green color was determined at 620 nm against a blank containing only water and anthrone reagent.

#### Nitrogen metabolism

Nitrogen metabolism was tracked by measuring the total nitrogen according to Lang (1958). 5 mg dry weight and 0.20 ml of 0.2 N H₂SO4. The acid digest is diluted to 10 ml. with water, and an aliquot of 3.0 ml. or less is transferred to another tube. To this is added water bringing the volume to 4.0 ml., and 2.0 ml. of Nessler reagent are then added. This reagent is prepared by dissolving 68 g of Nessler Granules (Tenso-Lab, Irvington-on-Hudson, N. Y.) in 100 ml. of water, followed by the additions of 850 ml. of an aqueous 10% sodium hydroxide solution and of water to a final volume of 1000 ml. After 10 min the developed color was measured at 420 nm. The contents of amino acids and proteins following the scheme of Mahmoud et al. (2023). Free amino acids were determined in the previous water extract of sugars. In clean empty test tube, add one ml of stannus chloride reagent to 0.5 ml of the water extract of plant samples. Boiling the test tubes in water bath for 20 min and then cooling. Add 4 ml of diluent solvent and mixed rapidly. The extinction of violet color was recorded spectrophotometrically at wavelength 570 nm against blank containing all the above reagents and distilled water instead of the extract of plant sample. A calibration curve was constructed using glycine and the data were expressed as mg amino acid (glycine)/ g D. W. For proteins 0.05 gm dry tissue was mixed with 10 ml NaOH (1 N) in test tubes, heated in boiling water bath for 1 h. Finally, this solution was cooled, filtered in sterilized vials and kept in refrigerator until use. 5 ml of the alkaline reagent solution were added to 0.1 ml of the water extract of plant samples. The tubes mixed and allowed to stand at room temperature for 10 min. Add 0.5 ml of diluted Folin Ciocalteau's reagent (1: 2 v/ v) and mixed rapidly. After 30 min., the extinction of blue color developed against appropriate blank was read at 750 nm. A calibration curve was constructed using egg albumin and the data were expressed as mg protein/ g DW.

Nitrate reductase activity (NR; mmolNO_2_/g/h) as described by Downs et al. (1993). Healthy, green, fully expanded leaves or fascicles were pinched from each plant were chopped into 2-mm squares and transported on ice to our laboratory. Prepared tissues were incubated in buffered (pH 7.0) 40 mM KNO3 with 1.5% propanol added to aid tissue infiltration. Enzyme activity was stopped by placing vials in boiling water. Aliquots of incubation buffer were analyzed colorimetrically for nitrite by adding 0.5 ml each of 1% sulfanilimide in 1:5 HC1 and 0.1% n-napthylethylene diamine dihydrochloride and measuring absorbance at 535 nm (Unico UV-2100 spectrophotometer).

#### Oxidative indicators and membrane damage attributes

Superoxide anion (μg/gFW, O_2_^·−^), hydroxyl radical (μmol/gFW, ^·^OH), and hydrogen peroxide (mg/g FW, H_2_O_2_) were analyzed through the technique of Abeed et al. (2021) as follows: The analysis of O_2_^·−^ in fresh materials was conducted by observing the nitrite accumulation from hydroxylamine at 530 nm employing a spectrophotometer (Jenway™ Genova spectrophotometer, Bibby Scientific, Staffordshire, UK). The content of ^·^OH in strawberry tissues was assessed in the reaction mixture comprised of 15 mM deoxyribose, 20 mM phosphate buffer (pH 7.4), 100 μM ferric chloride, 104 μM EDTA, 1 mM H_2_O_2_, and 100 μM ascorbate. At 37 °C, the extract was incubated for 1 h, followed by the addition of TCA and TBA and recognizing the final distinguished color at 532 nm via a spectrophotometer. The production of H_2_O_2_ was assessed by crushing fresh tissues (0.5 g) in chilled acetone (4 ml). Afterward, 3 ml of the extract and 1 ml of titanium dioxide (0.1%) were suspended in 20% sulfuric acid prior to centrifugation at 6000 rpm for 15 min. Then using a spectrophotometer, the formed yellow pigment of the reaction was checked at 415 nm using a standard curve. Lipid peroxidation was assessed as malondialdehyde (MDA; μmol g^−1^ FW) using the protocol of Rao and Sresty (2000) based on TBA (thiobarbituric acid) reaction. Fresh strawberry leaves were smoothed in 0.1% trichloroacetic acid (TCA) and then samples were placed in a centrifuge for 10 min at 10,000 rpm. Subsequently, the aliquot (1 ml) was added to TCA-TBA combination, and boiled at 90 °C for 30 min. After cooling, the samples were recentrifuged for 15 min at 10,000 rpm. Finally, The OD of the mixture was noticed at 532 nm by using a spectrophotometer and the measurements were adjusted for undefined turbidity via deducting the measurements at 600 nm. Electrolyte leakage (EL%) was estimated by conduct meter (Starlac Industries, Ambala, Haryana, India) according to Silveira et al. (2009). At 10 °C, fresh tissues were cut into small fragments and then saturated in 30 ml of deionized water. Primary electrical conductivity (C1) for the bathing solution was detected at 25 °C after 24 h of incubation. The secondary electrical conductivity (C2) was documented after heating samples in an autoclave at 121 °C for 15 min and allowing to cool down. The final EL% was calculated based on the variance between the secondary and primary by utilizing the presented equation: EL = (C1/C2) × 100.

#### Secondary metabolites

Proline measurement was analyzed as detailed by Bates et al. (1973). Tissues were added first to 3% sulfosalicylic acid and then to a mixture of proline: glacial acetic acid: acidic ninhydrin (1: 1: 1, v/v) before boiling at 100 °C for an hour. The reaction was then ended by transferring the test tubes to an ice bath. Finally, the mixture was extracted by toluene (2 ml) on a vortex. By exploiting toluene as blank, the OD of the developed organic part was acquired at 520using a spectrophotometer (Jenway™ Genova spectrophotometer, Bibby Scientific, Staffordshire, UK). Ascorbic acid (AsA) content was evaluated based on Jagota and Dani (1982) reported method. Briefly, plant tissues (300 mg) were emulsified in 2 ml of TCA (5%), and then centrifuged at 10,000 rpm (4 °C) for 15 min. Subsequently, the homogenate (200 μl) was added to TCA (0.8 ml, 10%). The reaction was then hindered by placing the samples in an ice bath for 5 min followed by re-centrifuging (3000 rpm) for 5 min. The diluted extract (500–2000 μl) was mixed with diluted Folin's reagent (200 μl). Mixture was incubated for 10 min and the amount of the developed blue pigment was evaluated at 760 nm using a spectrophotometer. To quantify flavonoid level in strawberry leaves, the methanolic extract of leaf samples was added to 5 ml distilled water and 3 ml AlCl_3_, then mixed with 2 ml CH_3_–COOK (1 M). The final absorbance of the homogenate was detected at 415 nm based on Harborne and Williams (1998) method. According to Kofalvi and Nassuth (1995), the Folin-Ciocalteu phenol reagent was used to determine the phenolic content. Methanol extract (100 μl) was diluted to 1 ml with distilled water and reacted with 0.5 ml of 2 N Folin-Ciocalteu's reagent and 2.5 ml of 20% Na_2_CO_3_. The mixture was left to cool down for 20 min at room temperature then the spectrophotometric readings were recorded at 725. The accumulation of reduced glutathione (GSH) in plant samples was evaluated by extracting the tissues in phosphate buffer (pH 8), then adding to 5, 5-dithiobis-2-nitrobenzoic acid. The samples wavelength was documented 412 nm using a spectrophotometer, and the concentration of GSH was explored from the calibration curve of GSH (Ellman 1959).The quantity of phytochelatins (PCs) in strawberry leaves was measured following the method of Nahar et al. (2016) by subtracting the measurement of GSH from the measurement of non-protein thiols which was achieved by integrating the supernatant of leaves ground in sulfosalicylic acid into Ellman’s reaction mixture as reported earlier (Ellman 1959).

##### Enzymatic antioxidant capacity

Following the technique of Abeed et al. (2022a) the analysis of enzymatic antioxidants was conducted. In brief, frozen leaf tissues (20 mg) were powdered with liquid nitrogen and then mixed with 3 ml of 100 mM potassium phosphate buffer (pH 7.8) involving 0.1 mM ethylenediamine tetraacetic acid (EDTA) and 100 mg polyvinylpyrrolidone. The mixture was centrifuged at 18,000 rpm for 10 min (4 °C) and then the supernatants were collected and employed for the specific enzymatic analysis. All colorimetric measurements were performed at 20 °C via UV spectrophotometer (Jenway™ Genova spectrophotometer, Bibby Scientific, Staffordshire, UK). The enzymatic capacity in strawberry leaves was explored by determining the specific activity of superoxide catalase (CAT; EC 1.11.1.6, U/mg protein/g FW/min) by observing the drop in OD at 240 nm for 1 min due to the interruption of H_2_O_2_. The enzyme activity was assessed in 4 ml medium having 4 components of 100 μl H_2_O_2_ (10 mM), 50 mM potassium phosphate buffer (pH 7), and 50 μl enzyme extract. Dismutase activity (SOD; EC.1.15.1.1, μmol/mg protein/g FW/min) was assessed by measuring the inhibition of the photochemical reduction in nitro-blue tetrazolium (NBT). The activity of 1 unit SOD was measured as the quantity of the enzyme that was considered to restrain 50% of the reaction in the absence of the enzyme for 1 min. The reads of the spectrophotometer were taken at 480 nm. Ascorbate peroxidase activity (APX; EC1.11.1.11, μmol/mg protein/g FW/min) was estimated according to the decline in absorbance at 290 nm for 1 min. Enzyme extract (50 μl) was poured into a tube containing the assay mixture, (0.5 mM ascorbic acid, 0.1 mM Na_2_-ETDA, 50 mM potassium phosphate buffer (pH 7), and 5 mM H_2_O_2_). The records were observed spectrophotometrically at 290 nm. The activity of glutathione peroxidase (GPX; EC.1.11.1.9, μmol/mg protein/g FW/min) was quantified by adding 0.2 ml of the supernatant to 0.4 ml of reduced glutathione, and then mixing 0.48 ml of the supernatant with a mixture involving 2.2 ml of 0.32 M Na_2_HPO_4_ and 0.32 ml of 1 mM 5, 5′-dithio-bis-(2-nitrobenzoic acid) till the appearance of the distinguished color. The absorbance at 412 nm was recorded with a spectrophotometer after 5 min. Glutathione-S-transferase (GST; EC 2.5.1.18, U/mg protein/min) activity was determined based on the reported records at 340 nm spectrophotometrically. Polyphenol oxidase (PPO; EC 1.10.3.1, μmol/mg protein/g FW/min) activity was considered by recording the alteration in OD reading at 495 nm. Then, five hundred ml of enzyme extract was combined with 0.1 M phosphate buffer (pH 6.0) and 0.1 M catechol. Lastly, 1 ml of sulfuric acid (2.5 N) was added to terminate the reaction. Phenylalanine ammonia-lyase (PAL; EC 4.3.1.5, μmol /mg protein/min) was measured by mixing the enzyme extract with the reagent mixture enclosing 25 mg of acetone powder, 1.5 ml of sodium borate buffer (100 mM, pH 8.0), and β-mercaptoethanol (20 mM). At 40 °C, the development of cinnamate for 2 h was measured by recording the OD alteration at 270 nm using a spectrophotometer.

#### Quality and nutritional status

The content of vitamin C in fruits was enumerated as mg ascorbic acid/100 ml juice by titration against 2,6- dichlorophenol-indophenol (A.O.A.C. 1995). Dye solution of 2,6- dichlorophenol-indophenol (0.025%) was prepared by dissolving 50 mg of it in 150 ml hot water containing 42 mg Sodium Bicarbonate and then filled to 200 ml with distilled water and stored at 3 °C. Dye molarity was determined by using 100 mg Ascorbic acid filled to 100 ml in volumetric flask with conservation solution (15 gm Oxalic acid + 40 ml Acetic acid 10% filled to 500 ml by distilled water). The titration with DCPI on 2 ml of conservation solution was made to adjust the dye molarity and then the titration with adjusted dye on 10 ml sample juice was executed to determine the vitamin C content.

Vitamin C (%) was calculated according to the following equation:

$$ {\text{V}}.{\text{C }}\left( \% \right) \, = \frac{{{\text{Dye }}\;{\text{volume }}\;{\text{used}}\;{\text{ in}}\;{\text{ titration}} \times {\text{Dye}}\;{\text{ molarity }}}}{{{\text{ sample}}\;{\text{ volume}}}} \times 100 $$

Total anthocyanin was determined as stated by Onayemi et al. (2006). Anthocyanins were determined after homogenizing of frozen fresh fruits in 10 ml acidified methanol (1% HCL v/ v).

The homogenate was centrifuged at 18,000 g for 30 min. at 4 °C and was stored in darkness for 5 h at 5 °C. The amount of anthocyanin was quantified using spectrophotometer at 550 nm, before anthocyanin measurement, background of the spectrophotometer was set using acidified methanol. The Folin-Ciocalteu (FC) reagent routine was employed to define the phenolics amount in the juice (Singleton and Rossi 1965). Fresh leaves (0.5 g) were extracted in 50% methanol (1: 1 v/ v) for 90 min. at 80 °C. The extract was centrifuged at 14,000 rpm for 15 min. free phenolics were determined in the supernatant using the Folin- Ciocalteu's phenol reagent. One hundred micro-liters of the methanol extract was diluted to 1 ml with water and mixed with 0.5 ml 2 N Folin- Ciocalteu's reagent and 2.5 ml of 20% Na_2_CO_3_. After 20 min. at room temperature, absorbance of samples was measured at 725 nm with Unico UV- 2100 spectrophotometer. By utilizing 1,1-diphenyl-2-picrylhydrazyl (DPPH), antioxidative activity was considered (Hussein et al. 2024). First, 100 µL of juice was diluted with 10 mL of a 6:4 (v/v) mixture of methanol and water (final ratio of juice/diluted methanol 1:100). Then, the diluted juice was combined with 2 mL of 0.1 mM DPPHin methanol, shaken, and left in the dark at room temperature for 15 min., The absorbance of the solution was measured at 515 nm with a spectrophotometer (Unico UV-2100 spectrophotometer). The reaction mixture without DPPH was used to correct the background. The antioxidant activity was computed using the following formula: antioxidant activity (%) = [1–(Abssample/Abscontrol)] 100. For nutrient analysis of Fe and Al, the samples were incubated at 70 °C and then digested using the wet digestion procedure (H_2_SO_4_-H_2_O_2_) (Parkinson and Allen 1975). Fe content in juice was measured spectrophotometry by Phenanthroline Method according to Pyenson and Tracy (1945). To Prepare Phenanthroline solution 300 mg 1,10-phenanthroline monohydrate, C12H8N2.H_2_O, were dissolved in 100 ml H2O. This reagent is sufficient for more than 300 μg Fe. Samples were prepared by mixing with 2 mL conc HCl and 1 mL NH2OH.HCl solution and heat to boil. Continue boiling until the volume is reduced to 15 to 20 ml to ensure complete iron dissolution (if the sample is ashed, take up residue in 2 ml conc HCL and 5 ml water.) Allow to cool to room temperature before transferring to a 50- or 100-ml volumetric flask. Dilute to mark with water 10 mL NH4C2H3O2 buffer solution and 4 mL phenanthroline solution. Allow at least 10 min for color development after thoroughly mixing. Light intensity is measured using a spectrophotometer with a wavelength of 510 nm. Al extraction was performed as illustrated in detail by Holak et al. (1980) and the content of Al was measured by using atomic absorption spectrophotometry.

#### Data analysis

All data are demonstrated as means ± standard deviation (SD) of four duplications. The comparisons among all groups for each treatment were identified statistically using the analysis of variance (ANOVA) through SPSS 21.0 software (SPSS Inc., Chicago, USA). Mean values for the treatments were compared, exploiting Duncan’s multiple range test (*p* < 0.05). Principal component analysis (PCA) was applied to investigate the multivariate structure of the dataset and to reveal the underlying correlations among the illustrating parameters. PCA was designed by employing JMP Pro® 6 (SAS Institute Inc., Cary, NC, USA). Multivariate classification was conducted by using PAST software v.2.11 (Hammer and Harper 2011).

Heatmap was generated applying R-Core-Team includes the (pheatmap) package and function. The mean was taken out of each result and divided by the standard deviation to standardise the data. As a result, all qualities will have a mean of zero and a standard deviation of one, making them comparable. Due to the different measuring units used by the analysed parameters, standardisation was conducted.

## Results

### Melatonin boosts strawberry growth and yield attributes under Al stress

As presented in Table 1, throughout the experimental period, Al stress markedly declined all growth-related parameters such as the root dry weight, crown dry weight, shoot dry weight, and leaf number by 22.7%, 31.3%, and 16%, respectively, compared to Al-untreated strawberries. In addition, the same decreasing trend was noticed in yield traits including fruit number, fruit weight, and fruit yield by 30%, 28.2%, and 48.5%, respectively. Compared with Al alone treatment, 50 ppm melatonin evidently modulated Al-negative impacts when it enhanced the root dry weight, crown dry weight, shoot dry weight, and leaf number in Al-stressed plants by 70.6%, 186.4%, 61.2%, and 116%, respectively, while clearly increasing fruit number, fruit weight, and fruit yield by 101.4%, 19.6%, and 214.9%, respectively. Furthermore, plants foliar sprayed with 100 ppm melatonin showed higher significant improvement in root dry weight, crown dry weight, shoot dry weight, and leaf number by 105.9%, 254.4%, 80.6%, and 152%, respectively, compared to free Al plants. Additionally, 100 ppm melatonin greatly boosted fruit number, fruit weight, and fruit yield by 14.4%, 84.3%, and 335.4%, respectively.

**Table 1:** Influence of melatonin (M0 = 0, M1 = 50, and M2 = 100 ppm) on growth parameters and yield analysis of strawberry plants grown under aluminum (Al) stress

Parameters	Treatments			
	Stress	M0	M1	M2
Root dry weight (g)	Al–	2.2 ± 0.2^c^	3.2 ± 0.1^b^	4.0 ± 0.03^a^
	Al +	1.7 ± 0.2^d^	2.9 ± 0.1^c^	3.5 ± 0.15^b^
Crown dry weight (g)	Al–	3.2 ± 0.05^d^	7.8 ± 0.06^b^	9.1 ± 0.21^a^
	Al +	2.2 ± 0.03^e^	6.3 ± 0.1^c^	7.8 ± 0.02^b^
Shoot dry weight (g)	Al–	8.0 ± 0.02^d^	11.8 ± 0.05^b^	13.3 ± 0.09^a^
	Al +	6.7 ± 0.1^e^	10.8 ± 0.7^c^	12.1 ± 0.04^b^
Leaf number (per plant)	Al–	8.2 ± 0.1^e^	11.9 ± 0.8^c^	13.1 ± 0.1^a^
	Al +	5.0 ± 0.03^f^	10.8 ± 0.1^d^	12.6 ± 0.08^b^
Fruit number (per plant	Al–	10.0 ± 0.05^e^	15.7 ± 0.09^c^	17.1 ± 0.3^a^
	Al +	7.0 ± 0.3^f^	14.1 ± 0.07^d^	16.9 ± 0.03^b^
Average fruit weight (g/plant)	Al–	7.1 ± 0.02^c^	8.9 ± 0.03^b^	10.1 ± 0.03^a^
	Al +	5.1 ± 0.04^e^	6.1 ± 0.1^d^	9.4 ± 0.07^b^
Fruit yield (g/plant)	Al–	70.4 ± 0.5^d^	140.2 ± 2.4^b^	173.7 ± 3.1^a^
	Al +	36.2 ± 0.3^e^	114.0 ± 0.6^c^	157.6 ± 1.1^b^

Means and standard errors of four independent replicates (n = 4). Diverse alphabetical letters donate significant variances among the treatments at *P* < 0.05

### Melatonin-modulated impacts on photosynthesis under Al stress

**Fig. 1:**
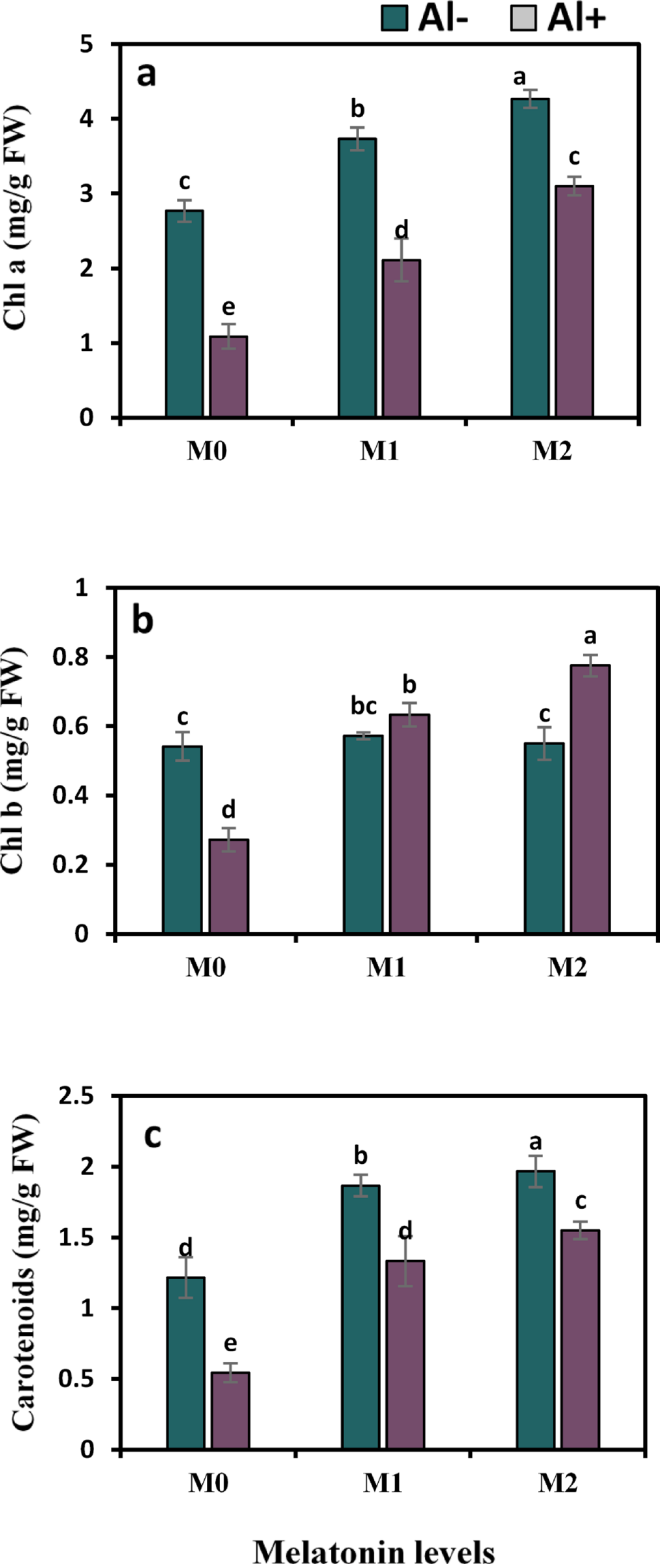
Influence of melatonin (M0 = 0, M1 = 50, and M2 = 100 ppm) on chlorophyll a (Chl a) (**a**), chlorophyll b (Chl b) (**b**), and carotenoids (**c**) of strawberry plants grown under aluminium (Al) stress. Bars show means and standard errors of four independent replicates (n = 4). Diverse alphabetical letters donate significant variances among the treatments at *P* < 0.05

Al imposed unfavorable influences on photosynthetic pigments in comparison to control plants, as shown by a substantial decline in Chl a, Chl b, and carotenoids by 60.6%, 50%, and 55.7%, respectively. However, the addition of 50 ppm melatonin to Al-stressed plants triggered the attributes mentioned above to remarkably elevate by 93.6%, 133.3%, and 146.3%, respectively. While the use of 100 ppm melatonin resulted in larger significant elevation of Chl a, Chl b, and carotenoids by 184.4%, 188.9%, and 187%, respectively (Fig. 1a–c).

### Impact of melatonin on carbohydrate assimilation under Al stress

**Fig. 2:**
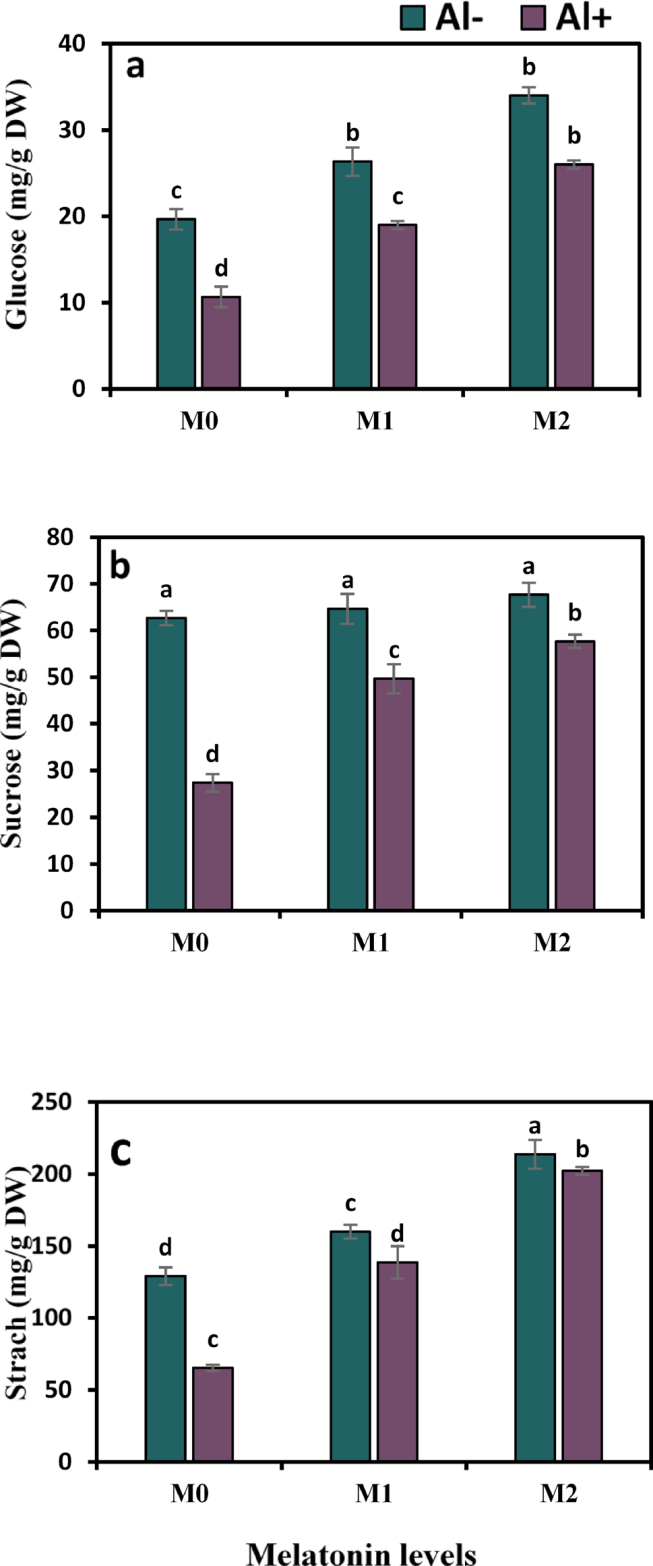
Influence of melatonin (M0 = 0, M1 = 50, and M2 = 100 ppm) on carbohydrate assimilation in terms of glucose (**a**), sucrose (**b**), and starch (**c**) contents of strawberry plants grown under aluminum (Al) stress. Bars show means and standard errors of four independent replicates (n = 4). Diverse alphabetical letters donate significant variances among the treatments at *P* < 0.05

Carbohydrate assimilation as measured by the contents of glucose, sucrose, and starch was negatively influenced by Al treatment, as displayed in Fig. 2a–c. On the other hand, there was a consistent increasing trend in these measurements by increasing melatonin dosage. In respect to control, Al declined glucose, sucrose, and starch values by 45.8%, 56.4%, and 49.4%, respectively. While in the joint treatment of Al + 50 ppm melatonin, the cellular content of glucose, sucrose, and starch profoundly increased by 78.1%, 81.7%, and 112.3%, respectively, whereas a greater increase of 143.7%, 110%, and 209.7%, respectively, was achieved when plants were subjected to Al + 100 ppm melatonin treatment.

### Influence of melatonin and Al stress on nitrogen metabolism

**Fig. 3:**
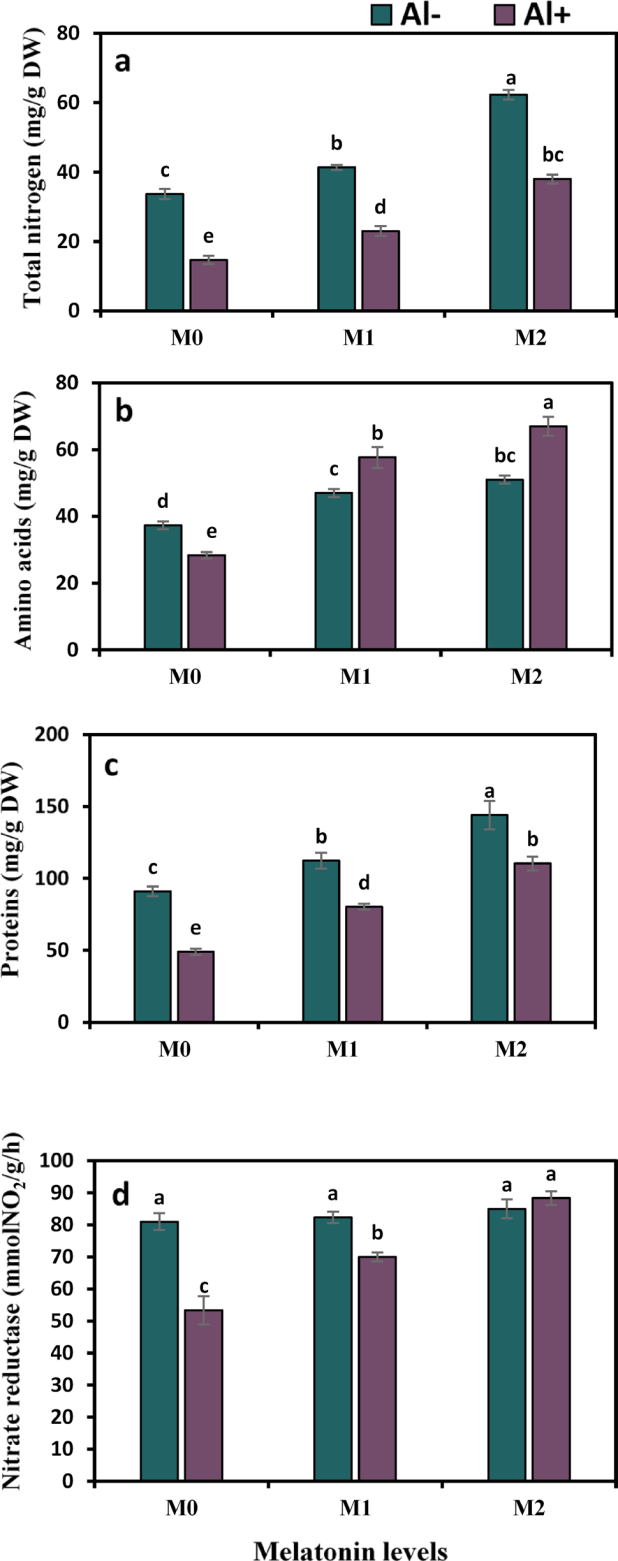
Influence of melatonin (M0 = 0, M1 = 50, and M2 = 100 ppm) on nitrogen metabolism in terms of total nitrogen (**a**), amino acids (**b**), proteins (**c**) contents, and nitrate reductase activity (**d**) of strawberry plants grown under aluminum (Al) stress. Bars show means and standard errors of four independent replicates (n = 4). Diverse alphabetical letters donate significant variances among the treatments at *P* < 0.05

Nitrogen metabolism was greatly disturbed under Al stress throughout the experimental period. Strawberry plants exposed to Al stress revealed lower rates of total nitrogen, amino acids, protein, and nitrate reductase by 56.4%, 24.1%, 46.2%, and 34.2%, respectively, than those of the control. By contrast, plants treated with 50 ppm melatonin under Al stress showed positive inclines in these parameters by 36.2%, 50.9%, 63.9%, and 31.3%, respectively, over their control. Moreover, the addition of melatonin at the maximal concentration (100 ppm) was more effective treatment for increasing these values by 159%, 136.5%, 125.2%, and 65.6%, respectively (Fig. 3a–d).

### Melatonin suppresses Al-induced oxidative damage

**Fig. 4:**
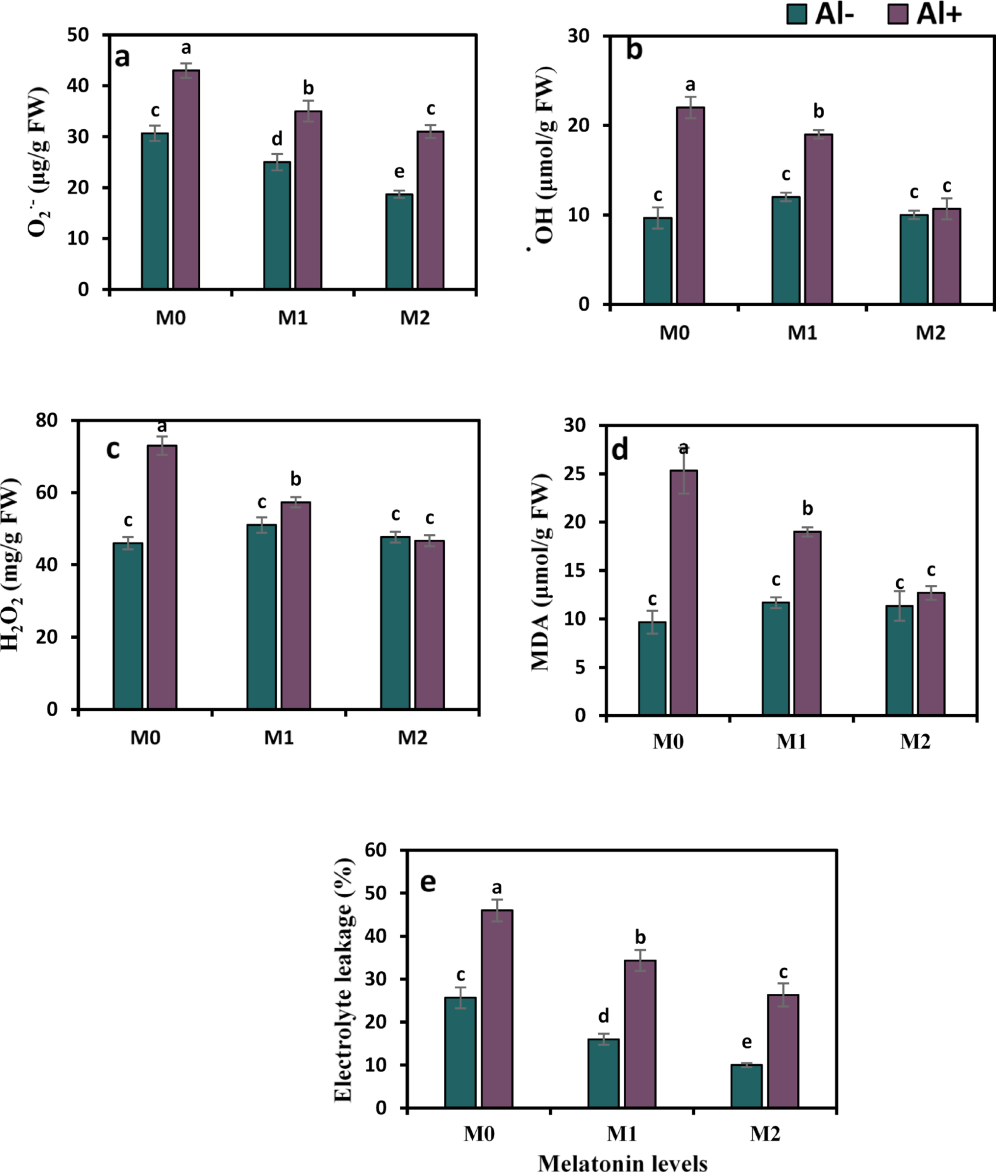
Influence of melatonin (M0 = 0, M1 = 50, and M2 = 100 ppm) on oxidative indicators and membrane damage attributes in terms of superoxide anion (O2·−) (**a**), hydroxyl radical (·OH) (**b**), hydrogen peroxide (H_2_O_2_) (**c**), malondialdehyde (MDA) (**d**) contents, and electrolyte leakage (%) (**e**) of strawberry plants grown under aluminum (Al) stress. Bars show means and standard errors of four independent replicates (n = 4). Diverse alphabetical letters donate significant variances among the treatments at *P* < 0.05

Stress markers are an important indication of the oxidative status under external stress. As shown in Fig. 4a–e, Al toxicity markedly triggered oxidative stress markers and membrane damage traits, mounting the levels of H_2_O_2_, O_2_^·−^, ^·^OH, MDA, and electrolyte leakage by 1.4, 2.3, 1.6, 2.6, and 1.8 times, respectively, versus those in control pots. These negative effects were obviously declined by 18.6%, 13.6%, 21.5%, 24.9%, and 25.4%, respectively, upon the application of melatonin (50 ppm) and by 27.9%, 51.4%, 36%, 49.8%, and 42.8%, respectively, under the concentration of 100 ppm in comparison to Al-treated plants with no melatonin applied.

### Melatonin empowers the activity of secondary metabolites

**Fig. 5:**
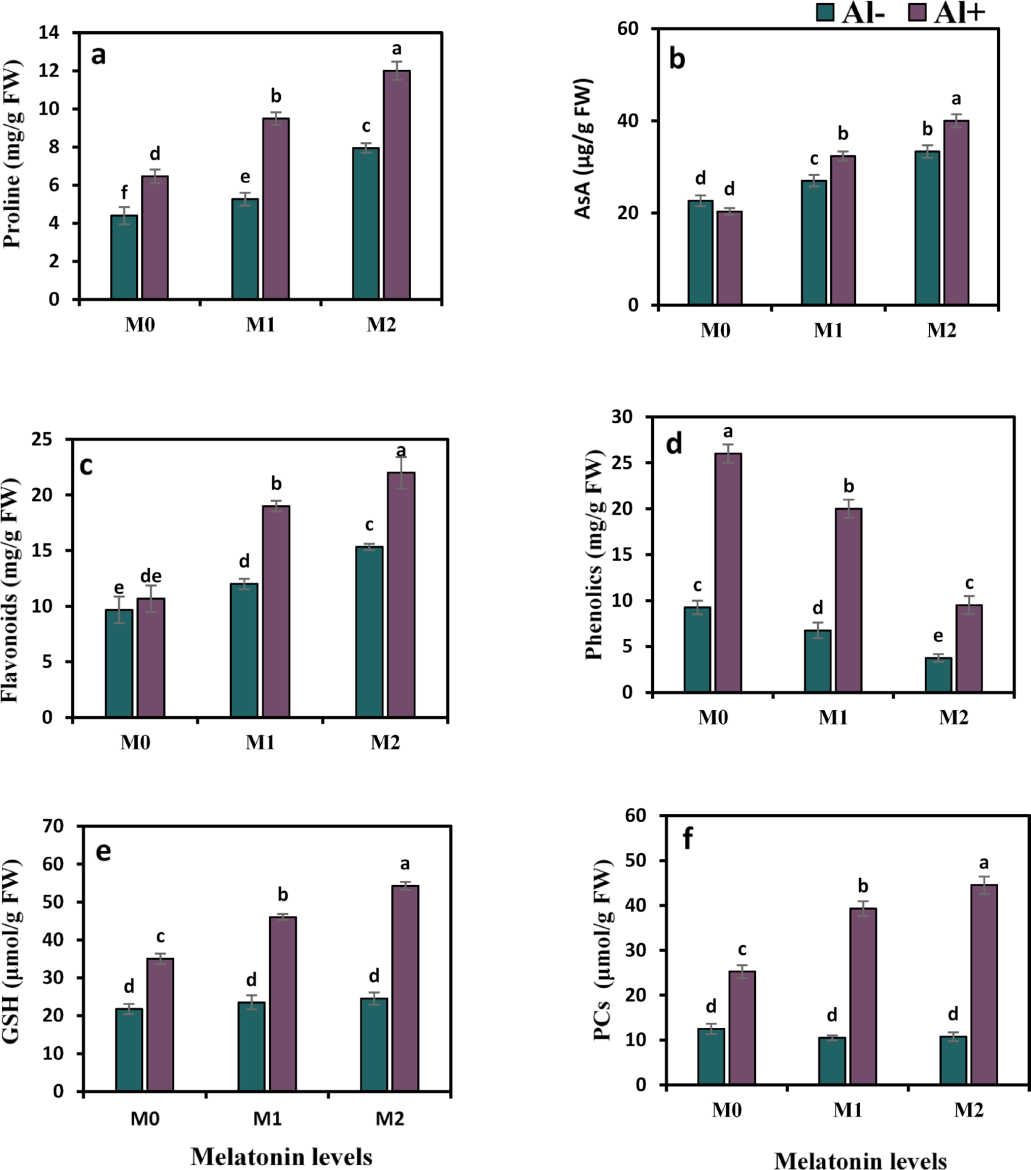
Influence of melatonin (M0 = 0, M1 = 50, and M2 = 100 ppm) on secondary metabolites in terms of proline (**a**), ascorbic acid (AsA) (**b**), flavonoids (**c**), phenolics (**d**), reduced glutathione (GSH) (**e**), and phytochelatins (PCs) (**f**) contents of strawberry plants grown under aluminum (Al) stress. Bars show means and standard errors of four independent replicates (n = 4). Diverse alphabetical letters donate significant variances among the treatments at *P* < 0.05

The values of the secondary metabolites showed variable changes in response to Al and melatonin treatments (Fig. 5a–f). Six major secondary metabolites were screened in this experiment. Al stress apparently improved proline, phenolics, GSH, and PCs by 47.7%, 181.1%, 60.9%, and 102%, respectively, while the pool of AsA and flavonoid showed non-significant changes relative to the control treatment. The addition of melatonin to Al-stressed plants led to further enhancement in these parameters except for phenolics. Proline, AsA, flavonoids, phenolics, GSH, and PCs content were elevated by 46.7%, 59.1%, 78.1%, 31.4%, and 76.4% under 50 ppm melatonin and by 85.5%, 96.8%, 106.2%, 55%, and 80.2% in response to 100 ppm melatonin, relative to those in Al-contaminated soil. In contrast, phenolics remarkably decreased by 23.1% and 63.5% under 50 and 100 ppm, respectively.

### Melatonin activates the antioxidant enzyme activity in Al-stressed plants

**Fig. 6:**
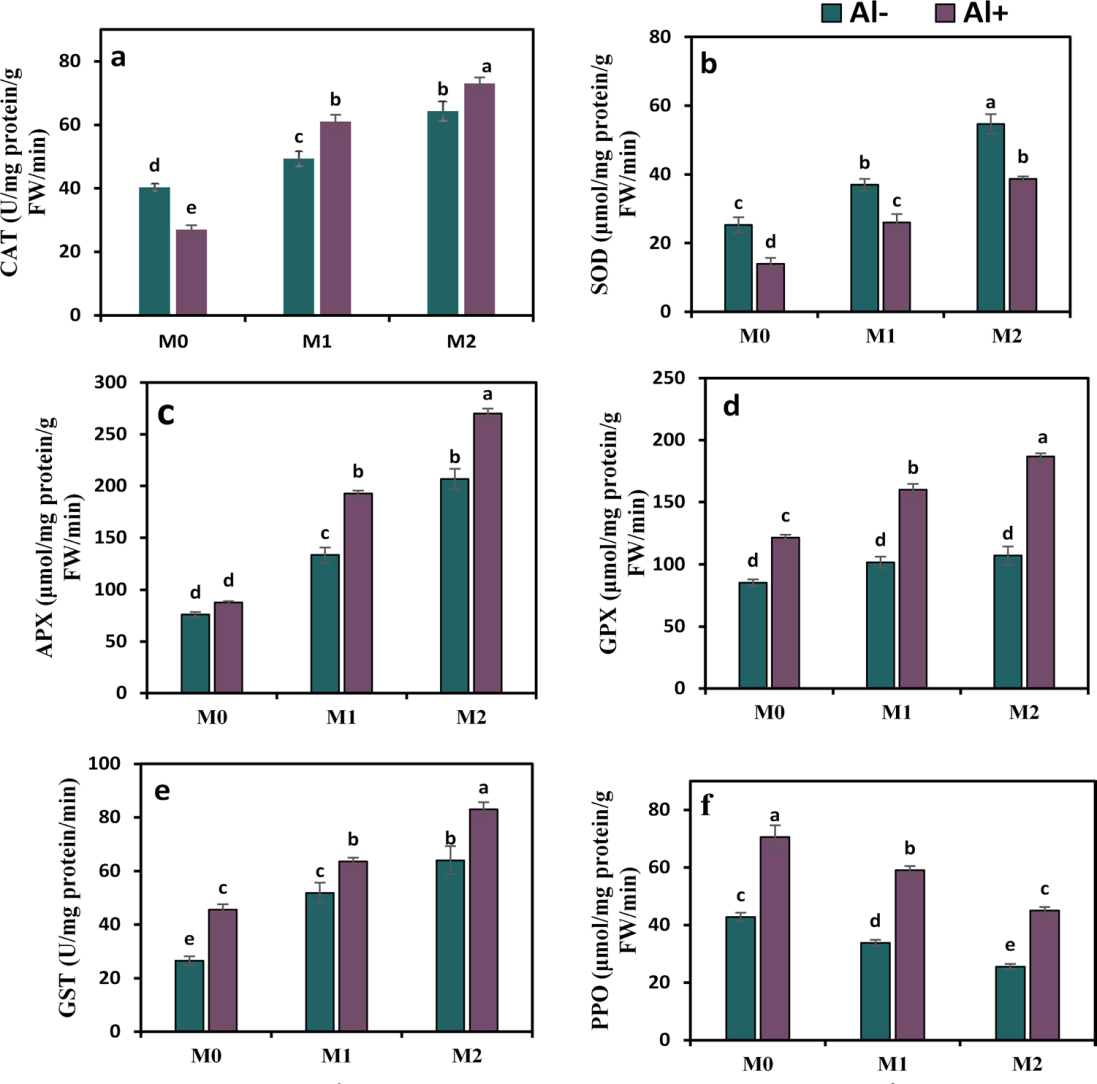
Influence of melatonin (M0 = 0, M1 = 50, and M2 = 100 ppm) on enzymatic antioxidant capacities in terms of catalase (CAT) (**a**), superoxide dismutase (SOD) (**b**), ascorbate peroxidase (APX) (**c**), glutathione peroxide (GPX) (**d**), glutathione-S-transferase (GST) (**e**), polyphenol oxidase (PPO), and phenylalanine ammonia-lyase (PAL) activities of strawberry plants grown under aluminum (Al) stress. Bars show means and standard errors of four independent replicates (n = 4). Diverse alphabetical letters donate significant variances among the treatments at *P* < 0.05

To evaluate the potential role of melatonin in improving tolerance in Al-stressed strawberry plants, alterations in the activity of enzymatic antioxidants were monitored in this investigation (Fig. 6a–g). Al declined the activities of CAT and SOD by 1.5 and 1.8-fold, respectively, compared to controls. While Al promoted the activities of GPX, GST, and PPO by 1.4, 1.7, and 1.6-fold, respectively. APX and PAL activities showed minor changes compared to control. The addition of melatonin together with Al reversed the impact of Al-induced oxidative disorders through the significant stimulation of CAT, SOD, APX, GPX, GST, and PAL activities by 2.3, 1.9, 2.2, 1.3, 1.4, and 1.7-fold, respectively, under 50 ppm melatonin and by 2.7, 2.8, 3.1, 1.5, 1.8, and twofold, respectively, under 100 ppm. Whereas PPO activity was dramatically decreased by 0.2 and 0.4-fold under the joint application of Al + 50 ppm melatonin and Al + 100 ppm melatonin, respectively.

### Fruit quality and nutritional value in response to melatonin and Al stress

Data in Table 2 exposed that Al had an adverse influence on vitamin C, anthocyanin, phenolics, antioxidants, and iron by 50.7%, 50.6%, 37.1%, 45.5%, and 23.7%, respectively, compared to the control. Supplementing the plants with melatonin had a pronounced effect and diminished the deleterious effects posed by Al. Treatment with 50 ppm melatonin remarkably raised vitamin C, anthocyanin, phenolics (%), antioxidants (%), and iron by 1.7, 1.5, 1.3, 1.4, and 1.1-fold over the.

**Table 2:** Influence of melatonin (M0 = 0, M1 = 50, and M2 = 100 ppm) on fruit quality and nutritional status of strawberry plants grown under aluminum (Al) stress

Parameters	Treatments			
	Stress	M0	M1	M2
Vitamin C (mg/100 mL)	Al–	27.2 ± 0.2^c^	34.4 ± 0.1^b^	41.6 ± 0.3^a^
	Al +	13.4 ± 0.1^e^	23.5 ± 0.1^d^	26.8 ± 0.2^c^
Anthocyanin (mg/100 g)	Al–	39.3 ± 0.3^c^	46.6 ± 0.3^b^	53.5 ± 0.4^a^
	Al +	19.4 ± 0.1^e^	29.5 ± 0.1^d^	37.5 ± 0.2^c^
Phenolics (%)	Al–	112.1 ± 0.6^b^	123.2 ± 0.7^a^	126.3 ± 0.5^a^
	Al +	70.5 ± 0.4^d^	94.2 ± 0.7^c^	106.3 ± 0.6^b^
Antioxidants (%)	Al–	79.3 ± 0.8^c^	92.2 ± 0.6^b^	118.3 ± 1.2^a^
	Al +	43.2 ± 0.3^e^	60.7 ± 0.5^d^	78.4 ± 0.8^c^
Fe (mg/kg)	Al–	3.8 ± 0.02^b^	4.3 ± 0.04^a^	4.8 ± 0.06^a^
	Al +	2.9 ± 0.04^d^	3.1 ± 0.06^c^	3.7 ± 0.02^b^
Al (µg/kg)	Al–	287.1 ± 1.5^d^	203.7 ± 1.0^e^	146.4 ± 0.5^f^
	Al +	642.8 ± 0.7^a^	382.9 ± 2.1^b^	330.3 ± 1.4^c^

Means and standard errors of four independent replicates (n = 4). Diverse alphabetical letters donate significant variances among the treatments at *P* < 0.05

Al-untreated ones. Plants given melatonin (100 ppm) had a higher content of these components under Al toxicity when they showed a notable increase of 2, 1.9, 1.5, 1.8, and 1.3-fold, respectively, in contrast to Al-stressed plants without melatonin. Significantly, melatonin at 50 and 100 ppm declined Al uptake and accumulation in strawberries by 40.4% and 48.6%, respectively.

### Principal component analysis

**Fig. 7:**
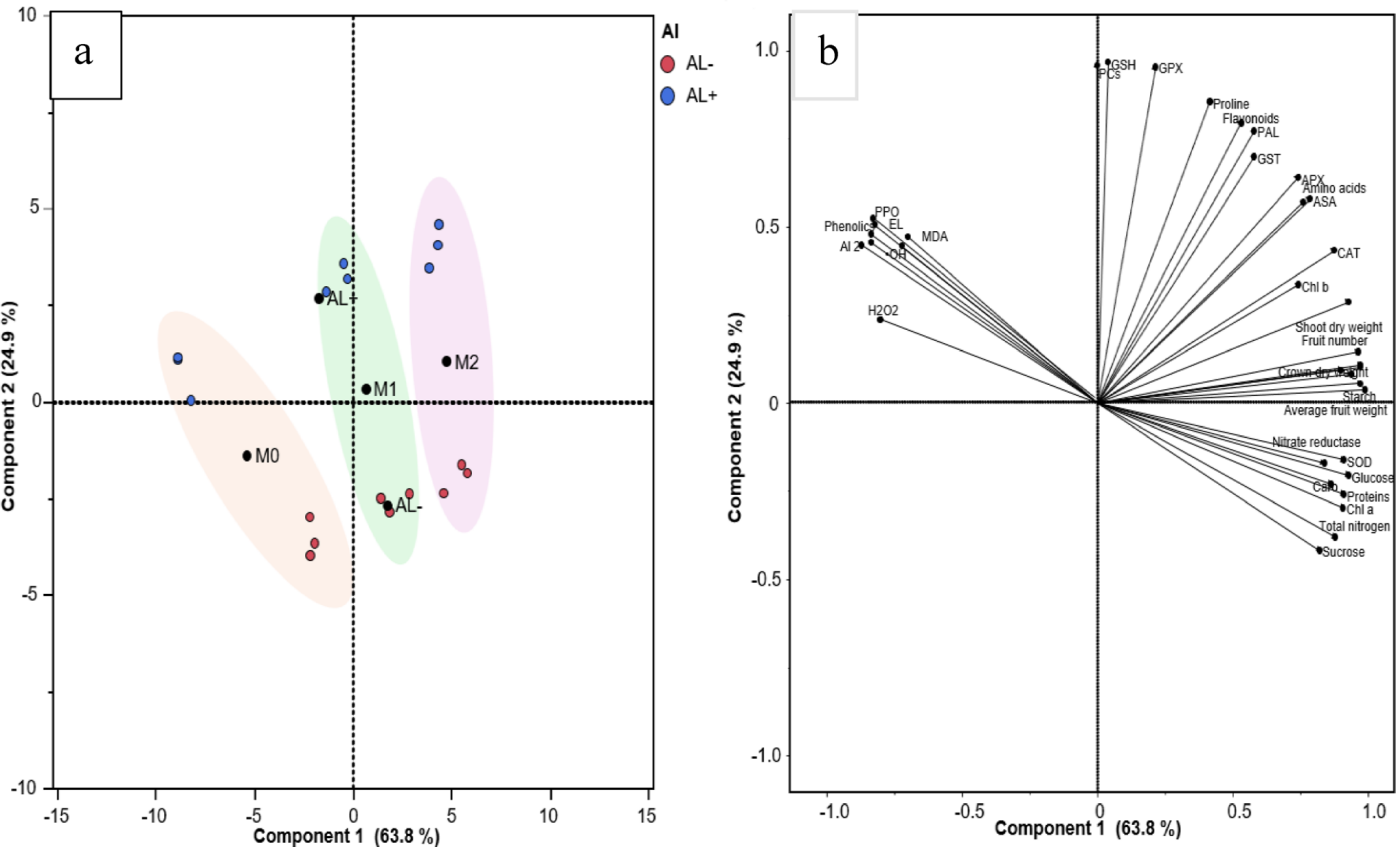
Principal component analysis (PCA) plot illustrating the influence of melatonin on the morphological and physiological responses of strawberry grown under aluminum (Al) stress

The principal component analysis (PCA) plot clearly highlights the morphological, physiological, and biochemical differences between the melatonin-treated and untreated plants under Al stress. The PCA analysis pattern displays an observed separation between the different treatments across two dimensions, with PC1 and PC2 explaining 63.8% and 24.9% of the variance, respectively. The definite separation of the treatments on the PCA plot underlines the considerable differences in their morphological and physiological responses. Al stress induces an apparent influence on strawberry plants, as shown in Fig. 7a and b. PC1 (63.8% of the variance) is mainly driven by growth and yield-related traits such as average fruit weight, shoot dry weight, fruit number, sucrose, chlorophyll, and total nitrogen, indicating its strong association with plant productivity. In contrast, stress markers like H₂O₂, MDA, and electrolyte leakage negatively influence PC1. PC2 (24.9%) reflects antioxidant and defense-related responses including GSH, GPX, proline, flavonoids, and PAL. These clarifications help explain how different treatments influence strawberry performance under Al stress.

### Multivariate analysis

**Fig. 8:**
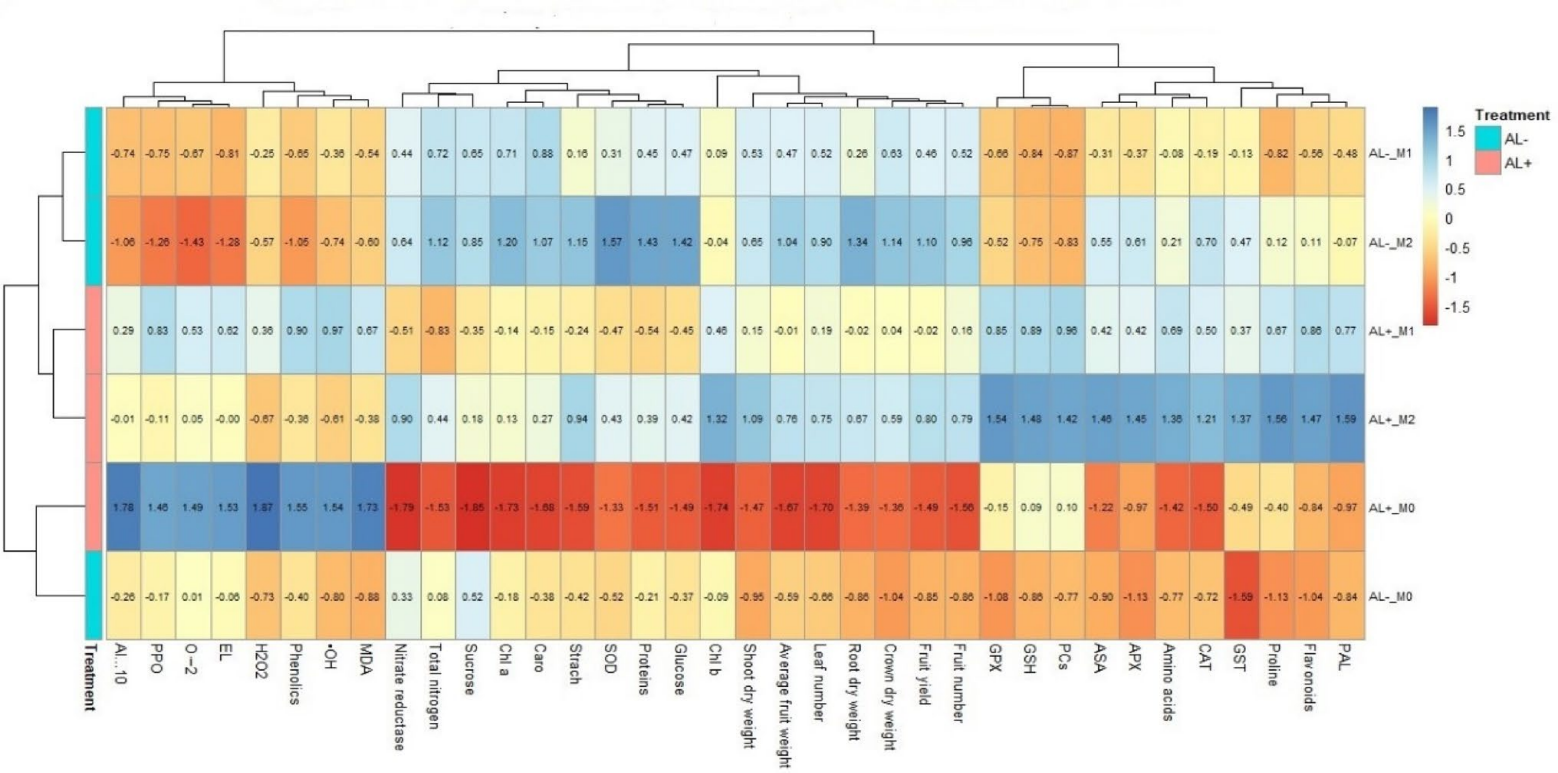
Multivariate cluster analysis of the strawberry plant morpho-physiological and biochemical attributes. Appreviations; SOD = superoxide dismutase, Chl a = Chlorophyll a, Caro = carotenes, GST = glutathione-S-transferase, ASA = ascorbic acid, CAT = catalse, APX = ascorbate peroxidase, PAL = phenylalanine ammonia-lyase, Chl b = Chlorophyll b, GSH = reduced glutathione, PCs = phytochelatins GPX = glutathione peroxide, Al = aluminum fruit content, O2·–= superoxide anion, EL = electrolyte leakage, PPO = polyphenol oxidase, ·OH = hydroxyl radical, MDA = malondialdehyde, H_2_O_2_ = hydrogen peroxide.Al−-M0 = without aluminum and 0 ppm melatonin, Al + -M0 = with aluminum and 0 ppm melatonin, Al−-M1 = without aluminum and 50 ppm melatonin, Al−-M2 = without aluminum and 100 ppm melatoinn, Al + -M1 = with aluminum and 50 ppm melatonin, Al + -M2 = with aluminum and 100 ppm melatonin

Multivariate classification (cluster analysis) expands the interpretation prosses for revealing the degree of similarity among the affected attributes. Thus, it conducted on the obtained data (Fig. 8) to understand all possible positive and negative correlations (similarities) among the plant morpho-physiological and biochemical traits.

Significant to highly significant correlations, either positive or negative, were achieved among the investigated plant attributes (Fig. 8). Plant traits were divided into 3 main groups. There were significant correlations between group A components including photosynthetic pigments (chlorophyll a and carotenes), growth and yield traits (root and shoot dry weight, leaf number, average fruit weight, fruit number, fruit yield, and crown dry weight), and carbon and nitrogen assimilation criteria (glucose, sucrose, starch, protein, total nitrogen, and nitrate reductase). These collectively express the morpho-physiological criteria. Whereas the biochemical criteria appeared in group B representing the antioxidant and metal detoxification potential of the cell including: glutathione-S-transferase, ascorbic acid, catalse, ascorbate peroxidase, amino acids, flavonoids, phenylalanine ammonia-lyase, chlorophyll b, reduced glutathione, phytochelatins, glutathione peroxide, and proline. These groups (A and B) recorded positive significant correlations (r^2^ = 0.5* to 0.8**) and showed significant negative correlation (r^2^ = -0.6*) with the third group of some toxic biochemical components (group C), which included aluminum fruit content, superoxide anion, electrolyte leakage, polyphenol oxidase, phenolics, hydroxyl radical, malondialdehyde, and hydrogen peroxide.

### Hierarchical clustering with heatmap

**Fig. 9:**
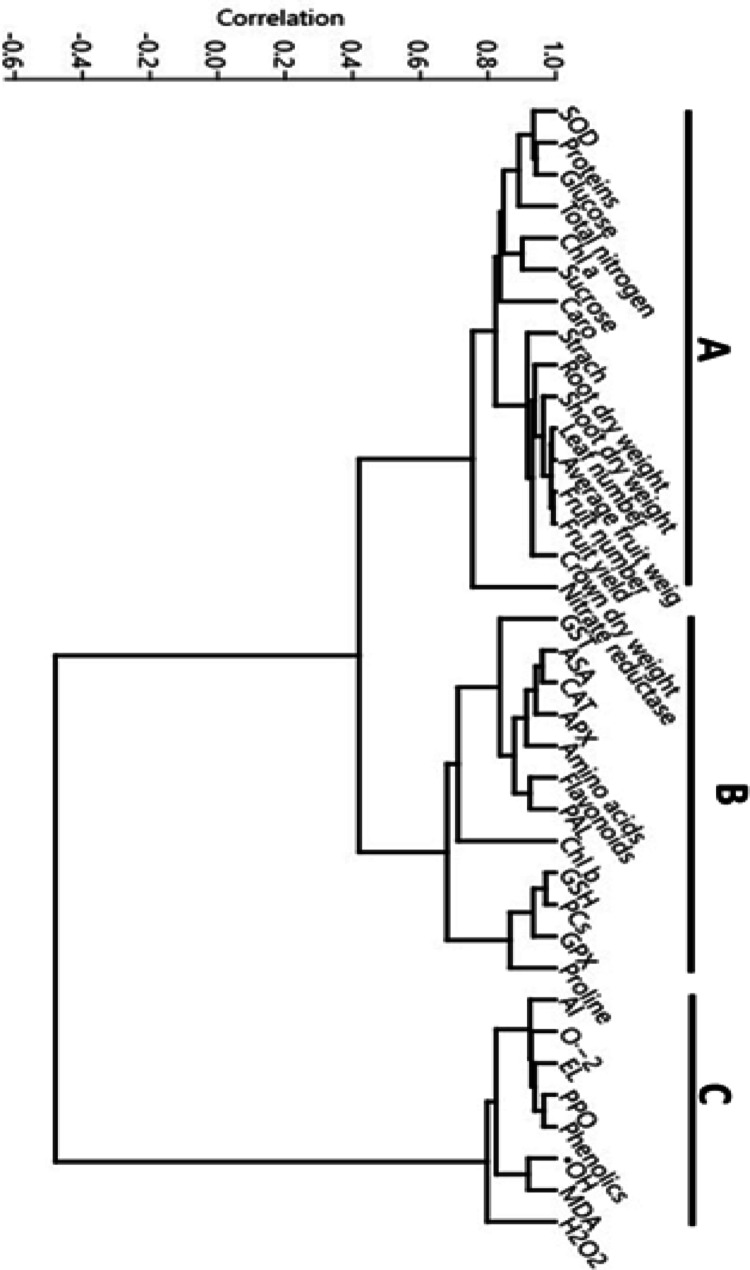
Heat map illustrating the normalized values of morpho-physiological and biochemical traits in strawberry plant across Al stress and treatment level combinations. Color intensity from light to dark denotes the range from low to high expression or activity levels, highlighting treatment-induced modulation under Al stress conditions. Appreviations; SOD = superoxide dismutase, Chl a = Chlorophyll a, Caro = carotenes, GST = glutathione-S-transferase, ASA = ascorbic acid, CAT = catalse, APX = ascorbate peroxidase, PAL = phenylalanine ammonia-lyase, Chl b = Chlorophyll b, GSH = reduced glutathione, PCs = phytochelatins GPX = glutathione peroxide, Al = aluminum fruit content, O2·–= superoxide anion, EL = electrolyte leakage, PPO = polyphenol oxidase, ·OH = hydroxyl radical, MDA = malondialdehyde, H_2_O_2_ = hydrogen peroxide.Al−-M0 = without aluminum and 0 ppm melatonin, Al + -M0 = with aluminum and 0 ppm melatonin, Al−-M1 = without aluminum and 50 ppm melatonin, Al−-M2 = without aluminum and 100 ppm melatoinn, Al + -M1 = with aluminum and 50 ppm melatonin, Al + -M2 = with aluminum and 100 ppm melatonin

**Fig. 10:**
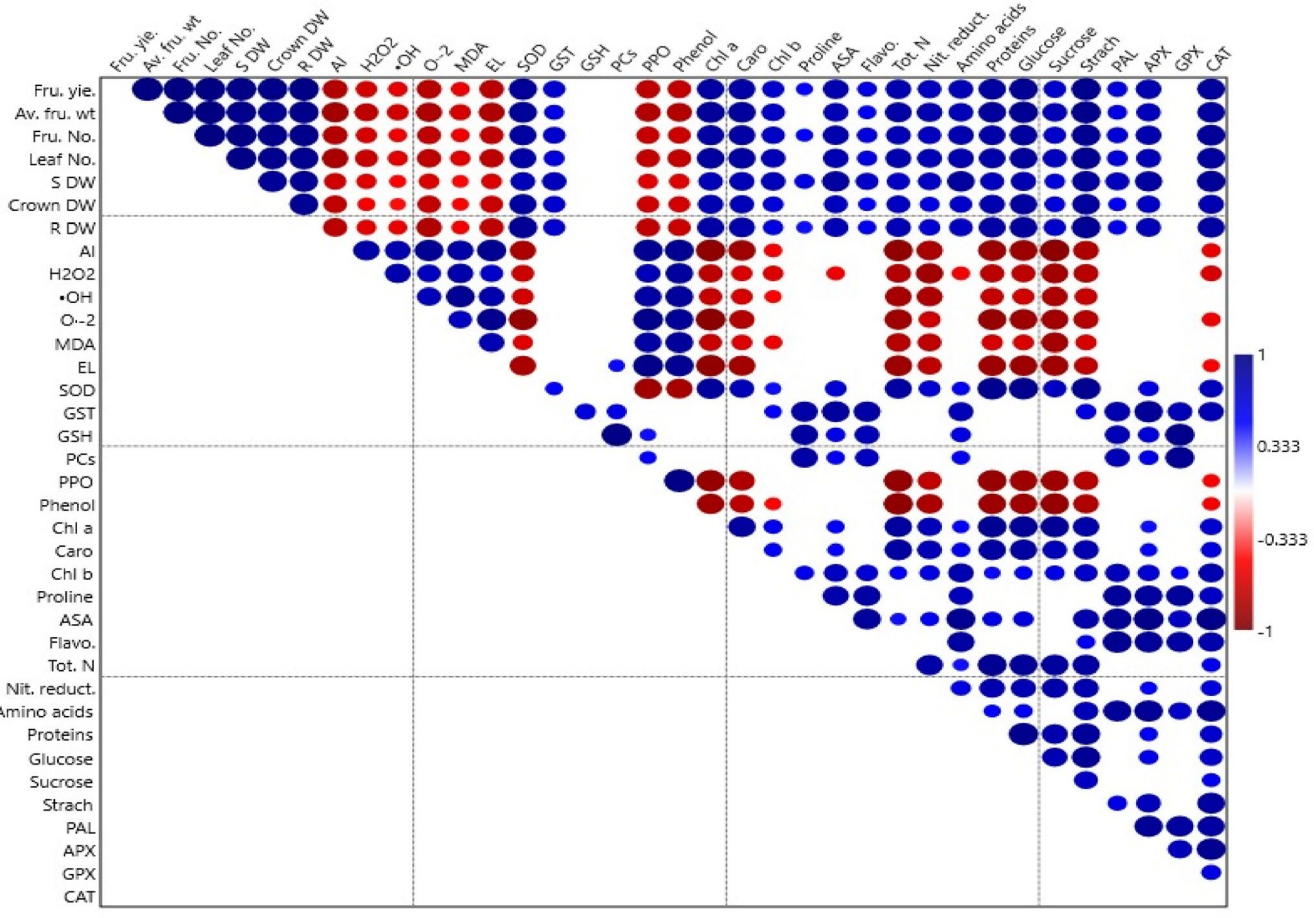
Pearson’s correlation coefficients among phenotypic, physiological, biochemical, and yield attributes of strawberry plant under Aluminum stress with treatments of melatonin. Positive correlations are represented in blue and negative correlations in red, with the size and intensity of the circles indicating the strength of the correlation

The heat map (Fig. 9) and the correlation matrix (Fig. 10) provide a comprehensive overview of the interrelationships among morphological, physiological, and biochemical parameters in strawberry plant subjected to Al stress, with and without mitigation by melatonin application. Strong positive associations were observed between plant growth traits (e.g. shoot, root and fruit biomass) and key physiological indicators. These parameters also exhibited significant positive correlations with antioxidants, suggesting enhanced physiological resilience linked to a robust antioxidative system in Al^−^-M0 treatment.

Remarkably, activity levels of enzymatic antioxidants also showed strong positive associations with both their respective substrates and other antioxidants, including PCs, GSH, phenolics, and flavonoids. This indicates a tightly regulated antioxidant defense mechanism operating at both transcriptional and biochemical levels thus showing positive correlations with plant performance indicators, highlighting the role of melatonin application in modulating stress responses and enhancing plant health under unfavorable conditions in case of Al^+^-M1 and Al^+^-M2.

Conversely, heavy metal stress indicators (Al accumulation in fruit) and oxidative stress biomarkers (e.g., H₂O₂, MDA, El) exhibited strong negative correlations with photosynthetic efficiency, antioxidant enzyme activities, and biomass accumulation. This negative trend indicates the detrimental effects of Al on plant metabolism and physiological function in case of Al^+^-M0. Furthermore, the increased activity of stress-responsive antioxidant enzymes coincides with enhanced phenotypic performance under Al unstressed condition combined with melatonin application (Al^−^-M1 and Al^−^-M2) submitted the systematic tolerance imparted by melatonin.

### Person’s analysis

Person’s analysis (Fig. 10) provides a comprehensive overview of the interrelationships among morphological, physiological, and biochemical parameters in strawberry plant subjected to Al stress, with and without mitigation by melatonin application. Strong positive associations were observed between plant growth traits (e.g. shoot, root and fruit biomass) and key physiological indicators. These parameters also exhibited significant positive correlations with antioxidants, suggesting enhanced physiological resilience linked to a robust antioxidative system in Al^−^-M0 treatment.

Remarkably, activity levels of enzymatic antioxidants also showed strong positive associations with both their respective substrates and other antioxidants, including PCs, GSH, phenolics, and flavonoids. This indicates a tightly regulated antioxidant defense mechanism operating at both transcriptional and biochemical levels thus showing positive correlations with plant performance indicators, highlighting the role of melatonin application in modulating stress responses and enhancing plant health under unfavorable conditions in case of Al^+^-M1 and Al^+^-M2.

Conversely, heavy metal stress indicators (Al accumulation in fruit) and oxidative stress biomarkers (e.g., H₂O₂, MDA, El) exhibited strong negative correlations with photosynthetic efficiency, antioxidant enzyme activities, and biomass accumulation. This negative trend indicates the detrimental effects of Al on plant metabolism and physiological function in case of Al^+^-M0. Furthermore, the increased activity of stress-responsive antioxidant enzymes coincides with enhanced phenotypic performance under Al unstressed condition combined with melatonin application (Al^−^-M1 and Al^−^-M2) submitted the systematic tolerance imparted by melatonin.

## Discussion

The toxicity of heavy metals is one of the greatest challenges for strawberry production and sustainability in polluted farmlands. Exposure to Al hinders the proper growth of plants and productivity. Nevertheless, the involvement of melatonin could have a regulatory role in combating Al stress. Melatonin initiates cascade of signaling defense responses through the activation of antioxidant network and the expression of defensive genes, leading to enhanced resilience during environmental adversities (Kolupaev et al. 2024) Fig. 11. Melatonin shares the same physiological roles as indole-3-acetic acid (IAA), since melatonin has the same precursor as IAA, thus it could elicit plant growth under stress (Siddiqui et al. 2019). In strawberry, melatonin significantly shielded plant performance and improved growth under cadmium stress (Wu et al. 2021; Saqib et al. 2023), iron deficiency (Kaya 2025), and drought (Khan et al. 2023). However, to the best of our knowledge, literature on the impact of melatonin enhancing Al tolerance in strawberry plants is scarce. In the present study, we explore the precise role of the melatonin in regulating strawberry growth and yield under Al exposure.

**Fig. 11:**
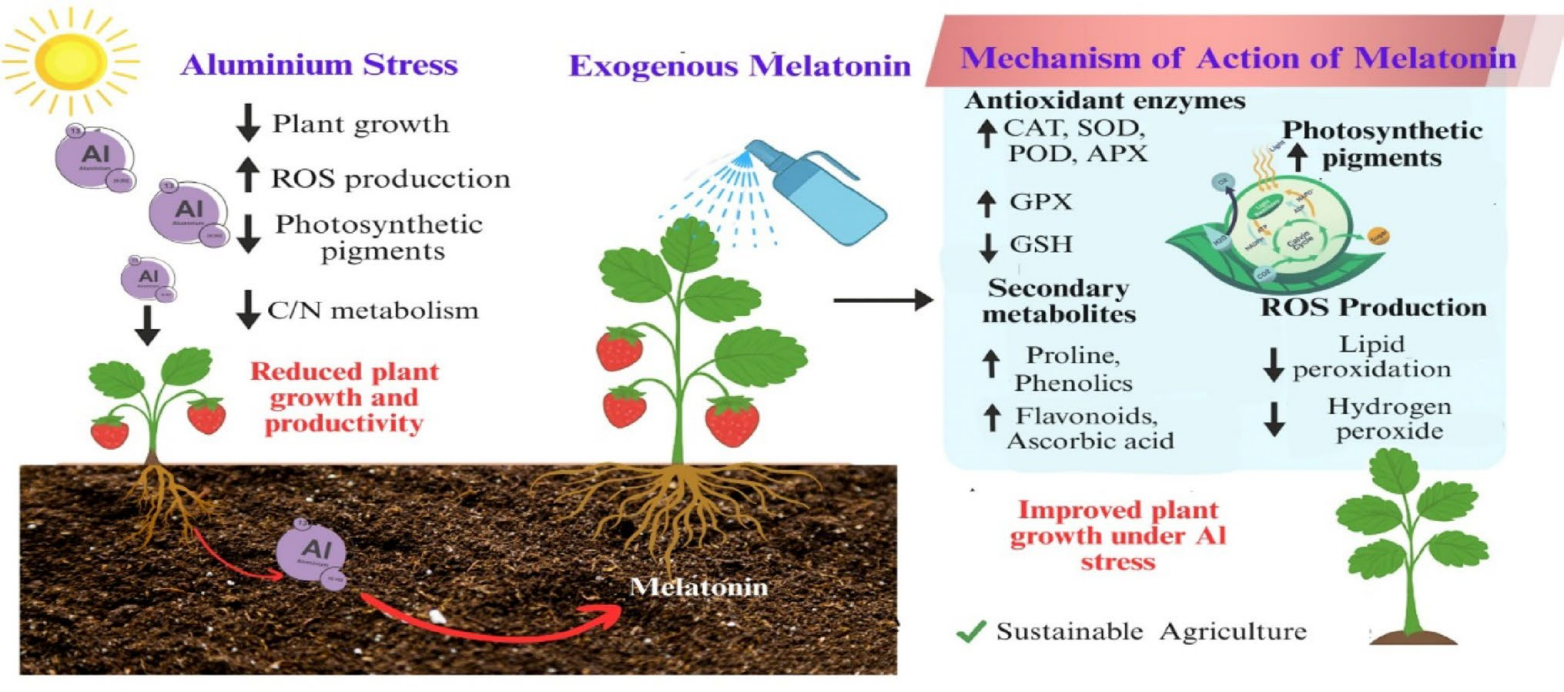
Mechanism of action of melatonin against AI with strawberry plant

Clearly, significant decreases in dry biomass, leaf number, and yield components (fruit number, fruit weight, and fruit yield) were observed in response to AL stress. The initial manifestation of Al toxicity in plants is the inhibition of plant growth as Al physically interacts with root system and causes Al uptake and migration to different plant organs such as shoot, leaf, and fruit, showing a reduction in growth and biomass (Al-Huqail et al. 2025). Root is a sensitive organ and root growth inhibition has been mainly used as an indicator in assessing Al toxicity in plants Chauhan et al. (2021). Al-induced restriction of root development by binding to the cell wall membrane, resulting in mechanical and functional destructions in root systems, which subsequently affects the overall growth (Sun et al. 2020a). The above accelerate conformational changes in biomolecules and instigate the uncontrolled accumulation of ROS, which interfere with cellular metabolism and suppress plant growth and yield (Singh et al. 2017). Melatonin was frequently reported to stimulate plant growth under heavy metal stress (Altaf et al. 2023; Yang et al. 2025a, b). Sun et al. (2020b) reported that increased melatonin level in wheat tissues alleviated Al-induced root growth inhibition to decrease Al stress. Exogenous application of melatonin was found to prompt the growth characteristics of maize by reducing Al accumulation in leaves and roots (Ren et al. 2022), which is crucial for achieving highest crop yields. In a similar study, 100 μM melatonin increased the total fruit yield and fruit average weight of strawberry by 41 and 47% under salinity stress, respectively, compared to control by modulating the antioxidant system (Zahedi et al. 2020). Zhang et al. (2014) reported that melatonin increased cucumber germination by regulating growth hormone signaling and the genes that incorporated in both ROS and plant hormone metabolism. Some investigations also observed that treating plants with melatonin counteracts the incline of MDA, declines electrolyte leakage, and preserves membrane stability (Hasan et al. 2015; Altaf et al. 2021b; Rizwan et al. 2024). These results corroborate the current study in that exogenous melatonin reduced the accumulation of Al in strawberry plants with greater effect at 100 ppm melatonin, thus restricting ion toxicity and cell membrane damage for achieving excellent plant growth and yield attributes.

Photosynthetic machinery is sensitive to metals hampering the chlorophyll biosynthesis (Khan et al. 2019). Mihailovic et al. (2008) stated that pigment content reveals the level of photosynthesis deterioration occurring under Al toxicity. It has been suggested that Al demolishes the photosystem by impairing chloroplast ultrastructure, distressing the electron transport chain, and disturbing ALAD enzyme; the precursor for plant pigments involved in photosynthesis. (De Sousa et al. 2022). In this research, Al harshly affected chlorophyll and carotenoid content compared to control. However, when melatonin was exogenously added to stressed plants, the contents of Chl a, Chl b, and carotenoids were quickly increased across boss levels, demonstrating the enhanced photosystem maintenance by melatonin. This result aligns with observations from other studies on melon (Zhang et al. 2017), tomato (Ghorbani et al. 2023), and fenugreek (Parwez et al. 2023), where the applied melatonin helped the plants to shield the photosynthesis activity during stress. Okatan et al. (2022) stated that melatonin increased the number of fruits and yield of strawberry due to increased leaf area and subsequently chlorophyll accumulation and photosynthesis rate. Therefore, the noted increases in chlorophyll levels in our study are not only linked to enhancing biomass production but also resulted in considerably higher yields. Sami et al. (2020) suggested the positive role of melatonin in motivating the enzymes implemented in the photosynthetic pathways, which led to controlling pigment degradation under Al stress. Melatonin is recognized to act as a potent antioxidant, modulating ROS generated under worrying conditions, including heavy metal stress (Liu et al. 2023). By controlling oxidative injury to chloroplasts and other cellular compartments, melatonin may contribute to the restoration of chlorophyll levels.

Al decreased the contents of carbohydrates, i.e., glucose, sucrose, and starch in strawberry plants, in harmony with values reported elsewhere in *Quercus serrata* (Moriyama et al. 2016). The synthesis of carbohydrates has a great contribution to the regulation of plant metabolism under stressful conditions (Hamed et al. 2025b). Carbohydrates have a key role in stabilizing membrane integrity and maintaining the cellular osmolality (El-Mahdy et al. 2018). Glucose is the principal integral of cell wall polysaccharides; reduced glucose contents can decline the cellular growth rate (Siddiqui et al. 2020). In our study, the decreased growth values align with those of declined carbohydrates observed during Al stress. In parallel, leaves are the primary source of biosynthesizing sucrose and starch, which are later loading to roots (sinks) to enhance the root growth (Song et al. 2020). Furthermore, when plants face stress, they remobilize stored carbohydrates to guarantee energy flow for essential metabolic activities when photosynthesis is imperfect (Thalmann and Santelia 2017). The notable increase in sugars and starch in the present study advocates that melatonin may improve primary metabolic pathway that is central for plant resilience and overall performance. In agreement, Ren et al. (2022) found a positive impact of melatonin in regulating sugar metabolism under Al stress, evidenced by 20.5% increase in sucrose content. Zhao et al. (2015) clarified that melatonin administrated plant activities under stress by promoting the transcription and activities of sucrose-phosphate and sucrose synthase, which are crucial enzymes for regulating sugar signaling. Ren et al. (2022) suggested that the rise in sucrose synthase transcript levels implies a probable accumulation of sucrose in alfalfa roots, denoting that melatonin may allocate more carbohydrates to the roots under Al toxicity. PCA revealed that there was noble agreement between increasing carbohydrate content and enhancing growth as well as photosynthesis dynamics.

N is an indispensable nutrient during plant growth and N deficiency influences crop growth performance. N is a key unit in proteins and amino acids, all of which are directly integrated in facilitating the biochemical and physiological functions in plant system (Anas et al. 2020). Synthesized amino acids are employed for the plant's own protein synthesis, thus boosting growth and crop yield (Trovato et al. 2021). Previous reports have declared that Al interrupts N assimilation by suppressing both N supply to the assimilation pathway and the activity of enzymes incorporated in N metabolism (Zhao and Shen 2018; Yang et al. 2024), as we observed in our work. Adequate plant growth and stress response largely depend on the tight regulation of photosynthesis and N assimilation. So, it is not surprising here that N metabolism deficiency might be the possible reason for decreased growth, chlorophyll, and carbohydrate content. In addition, we also observed a decreased activity of NR; the enzyme that interconverts NO_3_^−^ to NO_2_^−^ and serves as the rate-restricting enzyme for the entire nitrate assimilation pathway, which in general disrupts biological processes, vigorous growth, and yield output (Singh and Usha 2003). Hence, improved N status is an essential adaptive mechanism for plants to retain stress response under such stress. Notably, melatonin-treated plants exhibited an obvious modulation in N metabolism and NR enzyme activity. This implies the potential role of melatonin in regulating N metabolism, in line with similar outcomes noted in cucumber (Zhang et al. 2017), pepper (Kaya et al. 2022), and maize (Ren et al. 2022). The apparent increase in N assimilation had a lager influence on chlorophyll content, dry matter accumulation, and further yield increases. This metabolic regulation was confirmed by PCA analysis.

Al has adverse toxic effects on cellular membranes. Al was found to cause a heightened upraise in damage signaling molecules including free radicals (O_2_^·−^ and ^·^OH, H_2_O_2_) and MDA in plants (Malik et al. 2021), which consequently instigates membrane breakdown and raises electrolyte leakage (Fan et al. 2022). The uncontrolled provoke of ROS is one of the principal reasons of plant growth retardation under Al stress. PCA also showed negative correlation between stress markers and other morphological and physiological parameters. Advancing the antioxidant defense system can effectively reduce the oxidative symptoms induced by Al, and this is the role of melatonin in this work. Melatonin is a key regulator of heavy metal stress responses where oxidative stress is implicated, especially in Al response (Tu et al. 2025). Melatonin was reported earlier to serve as an MDA and H_2_O_2_ quencher (Sami et al. 2020). It has been proved that melatonin could increase the capacity of plants to scavenge H_2_O_2_, besides lessening the toxicity induced by H_2_O_2_ (Li et al. 2016). Moreover, our study conforms to multiple studies in those the presence of melatonin protects plasma membrane collapse and downregulates the oxidative stress induced by Al (Sun et al. 2020b; Ghorbani et al. 2023) and other heavy metals (Kaya et al. 2019; Ghorbani et al. 2024). In these studies, melatonin stimulates an advanced and efficient antioxidant defense network, competently cleansing harmful ROS like H_2_O_2_ and sustaining redox signaling balance. This finding supports the empowering activity of melatonin in defending versus Al stress via regulating the endogenous ROS levels.

A significant indication of plant adaptation to stress stimuli is the sufficient induction of secondary metabolites. Secondary metabolites are important non-enzymatic antioxidants emerge as first helpers in mitigating the adverse influences of increased toxicity in plants (Ashraf et al. 2018). Melatonin cannot directly hunt the free radicals. Melatonin has been frequently reported to quench the oxidative species by activating the secondary metabolism (Saqib et al. 2023). Simultaneously, we found that melatonin elicited the induction of proline, AsA, flavonoids, GSH, and PCs by several folds under Al stress, accompanied by an obvious decrease in the levels of O_2_^·−^,^·^OH, and H_2_O_2_. Secondary metabolites have a good hampering effect on Al-induced toxicity and oxidative harm through osmotic homeostasis and ROS intermediation mechanisms. AsA and flavonoids are powerful antioxidant compounds that can directly quench ROS in the cells (Elazab et al. 2021). Likewise, proline amino acid could protect cells against stress by regulating osmotic balance and ROS excessive formation (El-Mahdy et al. 2018). In accordance with our results, Farouk and Al-Amri (2019) documented that in rosemary plants treated with 50 μM melatonin and exposed to As stress, the increased production of proline (204%), AsA (5%), and flavonoids (65%) was a protective strategy to enhance As tolerance. Congruently, a recent investigation on sword lily also demonstrated that melatonin application (0.6 mM) increased the contents of flavonoids 135% and AsA 100% to suppress ROS negative impacts (Zulfiqar et al. 2024a, b). More recently, Chakraborty and Raychaudhuri (2025) found that melatonin application enhanced secondary metabolite (flavonoids and anthocyanins) pool by 2–threefold under Pb stress in *Plantago ovata*. Moreover, melatonin was suggested to be a metal-chelating agent (Wang et al. 2019) that has a potential capacity to detoxify heavy metals through the accumulation of chelators, viz., GSH and PCs, as we noted here. GSH also has a crucial role in the decrease of H_2_O_2_ to H_2_O. These results were similar to those reported in tomato and pepper (Hasan et al. 2015; Kaya et al. 2022), where GSH and PCs inhibited metal oxidation and decreased oxidative damage. These findings accentuate the importance of the secondary metabolites accumulation to protect strawberry plants against Al toxicity.

To tackle challenging ROS, plants employ diverse antioxidant defenses, primarily enriching the antioxidant enzyme pathway (Abeed et al. 2022a). Melatonin integrated in enzymatic regulation to curtail the downstream generation of ROS has been broadly reported. As observed in strawberry, CAT, SOD, and APX enzymes were decreased in Al-treated plants, revealing the poor defense system under Al stress. Whereas plants receiving melatonin corroborated greater CAT, SOD, and APX activities under the same conditions. SOD, the first line of defense against oxidative stress, has an essential role in converting O_2_^·−^ to O_2_ and H_2_O_2_, while CAT and APX are involved in downregulating ROS generation under heavy metal stress (El-Mahdy et al. 2025). Parallel outlines were noted in *Brassica napus* (Sami et al. 2020), strawberry (Saqib et al. 2023), and *Medicago sativa* (Liu et al. 2023), where these enzymes in coordination have the capability to track the free radicals and lower the ROS burst in stressed plants following melatonin treatment. Melatonin was also reported to modulate the activities of SOD, PPO, POX, CAT, and APX enzymes in response to salinity stress in *Phaseolus vulgaris* (Azizi et al. 2022). Among antioxidant defense mechanisms, GST plays a vital role in scavenging H_2_O_2_ by exploiting the action of glutathione as a substrate (Abeed et al. 2022b), while GPX effectively quenches noxious ROS. Consistent with multiple studies (Khan et al. 2020; Samanta et al. 2021), melatonin additive highly declined ROS levels in strawberry leaves by further motivating the activities of GST and GPX. This observation allies with the decreased oxidative stress markers followed by melatonin treatment in the current work. In tomato seedlings, melatonin moderated nickel-stress tolerance by upregulating the expression level of PAL (Jahan et al. 2020), matching with our results. Melatonin possibly activates PAL dynamics and declines PPO to modulate the secondary metabolites like flavonoids and AsA in turn, corroborating our results. Soluble sugars are signal molecules, contribute to organize the inner level of PAL activity (Jeandet et al. 2022). In parallel with our findings, Tousi et al. (2020) found positive correlation between the increase in soluble sugars and PAL activity, and PAL activity boosts the secondary metabolites i.e., flavonoids and anthocyanins contents in mallow plants under Cd stress. Moreover, Chakraborty and Raychaudhuri (2025) reported significant upregulation in the relative expression of genes (*PoPAL* and *PoPPO*) in melatonin treated- plants under Pb toxicity. Interestingly, the activity levels of enzymes at the dosage of 100 ppm melatonin were greater than those at 50 ppm, and this melatonin dose-specific effect was published by several authors (Sami et al. 2020; Wu et al. 2021; Li et al. 2022). In the process explained above, the properly regulated enzymatic mechanism operates synergistically with non-enzymatic one to shield critical biological processes like photosynthesis and stabilize redox homeostasis during Al stress. PCA also showed the significant and positive correlation between the antioxidant system and growth qualities.

Several studies have discussed the role of melatonin in enhancing fruit attributes during postharvest process, but rarely are known in response to metal stress. This study demonstrated that melatonin significantly improved the quality and nutritional status of strawberry fruits involving the contents of vitamin C, anthocyanin, phenolics, antioxidants, and iron. These compounds are key aspects of strawberry fruit quality and can behave as ROS scavengers. For instance, anthocyanins, a type of flavonoids, can control ROS overproduction and decline oxidative harm in collaboration with other antioxidants (Naing and Kim 2021). Melatonin enhances the fruit’s quality by adjusting broader metabolic pathways and limiting deleterious physiological effects on plants (Liu et al. 2016; Sadak et al. 2020). Shang et al. (2021) reported that melatonin elicited the accumulation of vitamin C, anthocyanin, phenolics, and antioxidants in blueberry fruits by upregulating the biosynthesis of antioxidant genes *VcAPX, VcGST,* and *VcPAL* while declining ROS accumulation, leading to overall fruit quality enhancement. These data and our data propose that this enhancement by melatonin could be related to the superior capacity of the antioxidant system, with reductions in oxidative injury. In the current work, the remarkable increase in the flavonoids content of melatonin-pretreated plants suggests that these compounds play an important role in modulating Al toxicity. Moreover, Fe is an essential nutrient for plant metabolism, and it was visibly increased by melatonin treatment, in line with increasing Fe (22.637%–151.503%) in *Platycladus orientalis* under Cd stress (Ou et al. 2023) as well as total and active Fe in peach (28.79% and 36.11%) under iron deficiency (Lin et al. 2022). The reason could be due to the similarity of structure and function between melatonin and auxins, which promoted the ferric-chelate reductase activity to regulate endogenous iron equilibrium as well as physiological processes under stress (Lin et al. 2022). Ahammed et al. (2020) demonstrated that melatonin regulated the expression levels of FRO2 and *IRT1* genes under low and high Fe content, signifying that melatonin has a key role in iron uptake. Moreover, Al toxicity interrupts mineral uptake in plants, leading to nutrient disruption and oxidative stress. By restricting Al absorbance, mineral uptake could be enhanced and promote iron accumulation in fruits. Notably, we observed that melatonin actively restricted the accumulation of Al in strawberry plants. Numerous studies have exhibited that melatonin proficiently inhibited the accumulation of Al and Cd in *Brassica napus* (Sami et al. 2020), Cd in *Fragaria ananassa* (Wu et al. 2021), and lead in *Plantago ovata* (Chakraborty and Raychaudhuri 2024) by regulating key genes involved in the ionic homeostasis and regulating the enzymatic and nonenzymatic systems. The decrease in Al accumulation had a great influence on declining ROS toxicity and improving plant growth and metabolism. These observations together assumed that melatonin, through various approaches, works as a stress-ameliorating agent and aids in modifying Al stress by upregulating the growth responses and metabolic pathways, as well as activating the ROS scavenging system, etc.

Ultimately, a straightforward multivariate correlation analysis condensed all of these studies, providing compelling data about the significance of melatonin implementation for Al-sensitive species (like strawberries) in order to overcome the phytotoxicity of this stress. To enhance data analysis, cluster analysis can group meters with similar characteristics (Abeed et al. 2023). Three clusters were created from the 36 qualities that were examined in this study. One cluster had hydrogen peroxide, aluminum fruit content, superoxide anion, electrolyte leakage, polyphenol oxidase, phenolics, hydroxyl radical, and malondialdehyde. This showed that maintaining membrane stability was associated with validation of the efficacy of melatonin therapies in reducing Al stress damages in the current investigation. One of the main causes of oxidative burst is the production of ROS, especially H_2_O_2_, which is a relatively long-persisted ROS. Changes in the amount of ROS that attack the cell membrane's phospholipid layer may be the cause of variations in the absorptivity of the membrane (MDA values can determine lipid peroxidation degree) (Abeed and Salama 2022). Therefore, the amount of MDA increases in proportion to their tolerance, negatively impacting membrane integrity and increasing cell leakiness. The abatement of both ROS and their toxic byproducts (oxidized proteins and lipid hydroperoxides) is a prerequisite for the survival of plants in the existence of toxic metals. This mechanism was activated by melatonin application. Existence of PPO and its substrates, phenolics in this group (C) advocated that PPO is related to stress conditions and involves the cell wall cross-linking and lignification process resulting in a reduction in cell wall extensibility, which restricts cell growth, revealing exhausted plant tissues (Abeed et al. 2022a, b, c). Melatonin treatment down-regulated this enzyme, which addressed proper growth and elongation under Al stress. Glutathione-S-transferase, ascorbic acid, catalse, ascorbate peroxidase, amino acids, flavonoids, phenylalanine ammonia-lyase, chlorophyll b, reduced glutathione, phytochelatins, glutathione peroxide, and proline were among the closely connected characteristics in the second cluster. According to the current study, melatonin's ameliorative effect may be attributed to its ability to increase the antioxidant properties (intercellular redox pool) of ASA, flavonoids, and chlorophyll b, as well as the detoxification power of high activated GST, CAT, APX, and GPX, as well as the complexation potential of GSH, PCs, and proline. These factors improved the photosynthetic efficiency of flourished chlorophyll a and carotenes, which in turn increased their membrane stabilizing activity. This further established that membrane dysfunction was the primary issue of the plant under Al stress. Furthermore, study demonstrated that the melatonin up-regulation function on the Al-stressed plant was linearly correlated with membrane stabilization. Pervasive and essential photosynthetic pigments, carotenoids and chlorophylls are closely linked to plant development indicators, such as plant and fruit dry weight, which indicate an enhanced mechanism for allocating carbon and nitrogen (Abeed et al. 2023). Their combined effect highlighted that the scavenging of ROS by increasing the capacities of ROS scavenger enzymes could also be linked to the effectiveness of melatonin treatments in enhancing plant performance and liveness, indicating that melatonin induced a metabolic defense strategy against oxidative stress and cell death driven by senescence (Zulfiqar et al. 2024a, b). Under such melatonin-mediated conditions, plants experienced reduced Al stress and focused their entire cell energy on improving the structure of their bodies by increasing the levels of proteins and starch (group A), rather than using these metabolites to scavenge radicals.

## Conclusion

The current research findings highlight the promising role of melatonin in mitigating Al toxicity in strawberry for sustainable agricultural practices. Applied melatonin remarkably boosted stress resilience in Al-stressed strawberry plants by enhancing biomass, photosynthetic pigments, and yield components. Melatonin alleviated the oxidative stress in strawberry leaves when declined the concentrations of MDA and stress markers as well as electrolyte leakage %. The foliar exposure to melatonin heightened the activities of antioxidant enzymes and secondary metabolites in the leaves, hence controlling the harmful ROS compounds in plant. Melatonin treatment led to the regulation of carbohydrates and nitrogen metabolism, which could play a role as crucial mechanism to shield strawberry plants from Al toxicity. Moreover, melatonin enriched the overall quality of the fruit by promoting the buildup of vitamin C, anthocyanin, phenolics, antioxidants, and iron. Melatonin also successfully lowered Al accumulation on fruits, further contributing to its role as a stress-ameliorating agent. Overall, the investigation may advocate that melatonin could effectually decline Al toxicity on strawberry, accordingly, lessening the potential health risks beyond fruit consumption for safe crop production.

## Data Availability

All data generated or analyzed for this study are included in this published article.
